# Preserving and enhancing mitochondrial function after stroke to protect and repair the neurovascular unit: novel opportunities for nanoparticle-based drug delivery

**DOI:** 10.3389/fncel.2023.1226630

**Published:** 2023-07-07

**Authors:** Robyn J. Novorolsky, Gracious D. S. Kasheke, Antoine Hakim, Marianna Foldvari, Gabriel G. Dorighello, Israel Sekler, Vidyasagar Vuligonda, Martin E. Sanders, Robert B. Renden, Justin J. Wilson, George S. Robertson

**Affiliations:** ^1^Department of Pharmacology, Faculty of Medicine, Dalhousie University, Halifax, NS, Canada; ^2^Brain Repair Centre, Faculty of Medicine, Dalhousie University, Halifax, NS, Canada; ^3^School of Pharmacy, Faculty of Science, University of Waterloo, Waterloo, ON, Canada; ^4^Department of Physiology and Cell Biology, Faculty of Health Sciences, Ben Gurion University, Beersheva, Israel; ^5^Io Therapeutics, Houston, TX, United States; ^6^Department of Physiology and Cell Biology, School of Medicine, University of Nevada, Reno, NV, United States; ^7^Department of Chemistry and Chemical Biology, College of Arts and Sciences, Cornell University, Ithaca, NY, United States; ^8^Department of Psychiatry, Faculty of Medicine, Dalhousie University, Halifax, NS, Canada

**Keywords:** stroke, mitochondrial calcium uniporter, sodium/calcium/lithium exchanger, retinoid X receptor, thyroid hormone receptor, neurovascular unit

## Abstract

The neurovascular unit (NVU) is composed of vascular cells, glia, and neurons that form the basic component of the blood brain barrier. This intricate structure rapidly adjusts cerebral blood flow to match the metabolic needs of brain activity. However, the NVU is exquisitely sensitive to damage and displays limited repair after a stroke. To effectively treat stroke, it is therefore considered crucial to both protect and repair the NVU. Mitochondrial calcium (Ca^2+^) uptake supports NVU function by buffering Ca^2+^ and stimulating energy production. However, excessive mitochondrial Ca^2+^ uptake causes toxic mitochondrial Ca^2+^ overloading that triggers numerous cell death pathways which destroy the NVU. Mitochondrial damage is one of the earliest pathological events in stroke. Drugs that preserve mitochondrial integrity and function should therefore confer profound NVU protection by blocking the initiation of numerous injury events. We have shown that mitochondrial Ca^2+^ uptake and efflux in the brain are mediated by the mitochondrial Ca^2+^ uniporter complex (MCU_*cx*_) and sodium/Ca^2+^/lithium exchanger (NCLX), respectively. Moreover, our recent pharmacological studies have demonstrated that MCU_*cx*_ inhibition and NCLX activation suppress ischemic and excitotoxic neuronal cell death by blocking mitochondrial Ca^2+^ overloading. These findings suggest that combining MCU_*cx*_ inhibition with NCLX activation should markedly protect the NVU. In terms of promoting NVU repair, nuclear hormone receptor activation is a promising approach. Retinoid X receptor (RXR) and thyroid hormone receptor (TR) agonists activate complementary transcriptional programs that stimulate mitochondrial biogenesis, suppress inflammation, and enhance the production of new vascular cells, glia, and neurons. RXR and TR agonism should thus further improve the clinical benefits of MCU_*cx*_ inhibition and NCLX activation by increasing NVU repair. However, drugs that either inhibit the MCU_*cx*_, or stimulate the NCLX, or activate the RXR or TR, suffer from adverse effects caused by undesired actions on healthy tissues. To overcome this problem, we describe the use of nanoparticle drug formulations that preferentially target metabolically compromised and damaged NVUs after an ischemic or hemorrhagic stroke. These nanoparticle-based approaches have the potential to improve clinical safety and efficacy by maximizing drug delivery to diseased NVUs and minimizing drug exposure in healthy brain and peripheral tissues.

## 1. Introduction

Stroke is the third leading cause of death and the tenth major contributor to disabilities requiring long-term care (Writing Group et al., 2016). Most strokes (85%) are ischemic and typically caused by a blood clot that blocks a major artery in the brain (Writing Group et al., 2016). The remaining strokes (15%) are hemorrhagic and result from the rupture of cerebral blood vessels (Writing Group et al., 2016). For those afflicted, their reduced quality of life and dependence on others also places a heavy burden on our society and healthcare networks (Anderson et al., 2004; Feigin et al., 2014). Based on data from a sample of high, middle- and low-income countries around the world, the annual treatment, rehabilitation, and indirect costs for stroke have been estimated to exceed $700 billion US dollars (USD) (Feigin et al., 2022). As the population continues to age, these costs are expected to be over $1 trillion USD by 2030 (Feigin et al., 2022). The development of drugs that protect the brain from damage and improve neurological recovery after a stroke would therefore mitigate a major burden on our society and heath care systems. The cost of developing a new drug is typically $10–15 billion USD (Wegener and Rujescu, 2013). Moreover, most therapeutic candidates for stroke fail only after late-stage clinical trials have been completed (Cheng et al., 2004; Choi et al., 2014). This has resulted in the closure of many drug discovery programs for stroke and a reluctance of pharmaceutical companies to invest the considerable funds required for the development of stroke therapeutics.

The NVU is the basic component of the blood brain barrier (Iadecola, 2004). This intricate structure is comprised of vascular cells, glia, and neurons that work in unison to rapidly adjust cerebral blood flow (CBF) in support of the dynamic metabolic demands imposed by neurotransmission (Muoio et al., 2014; Longden et al., 2016). Endothelial cells of the NVU form tight junctions and utilize a wide array of transporters that act as physical and biochemical barriers to oppose the accumulation of injurious metabolites and proteins in the brain (Loscher and Potschka, 2005; Iadecola, 2017). NVU to damage by a stroke therefore has devastating consequences for the brain (Schaeffer and Iadecola, 2021). Furthermore, aging, a risk factor for poor stroke outcomes (Shin et al., 2022), is known to suppress NVU repair (Cai et al., 2017). These findings highlight the paramount importance of preserving and repairing the NVU to effectively treat stroke.

The NVU responds to dynamic metabolic demands required for increased brain activity depends by rapidly altering CBF. The phenomenon, known as neurovascular coupling, depends heavily on mitochondrial Ca^2+^ uptake to rapidly buffer cytosolic Ca^2+^ and stimulate energy production in multiple cellular components of the NVU (Iadecola, 2017). However, this also renders the NVU highly susceptible to lethal mitochondrial Ca^2+^ loading (Halestrap, 2006; Duchen, 2012). Understanding how Ca^2+^ is handled by mitochondria thus has important therapeutic implications for the treatment of ischemic and hemorrhagic stroke.

To this end, we have shown that mitochondrial Ca^2+^ uptake and efflux in the brain are mediated by the mitochondrial Ca^2+^ uniporter complex (MCU_*cx*_) and sodium/Ca^2+^/lithium exchanger (NCLX), respectively (Palty et al., 2010; Nichols et al., 2017). These important findings led to our recent studies demonstrating that MCU_*cx*_ inhibition and NCLX activation potently protect neurons from ischemic/reperfusion injury (Nichols et al., 2018; Novorolsky et al., 2020) and excitotoxicity (Rozenfeld et al., 2022) thought to drive stroke-related brain damage (Choi, 1992; Eltzschig and Eckle, 2011). Based on these studies, we describe the combined use of drugs that block the MCU_*cx*_ and stimulate the NCLX to protect the NVU after an ischemic or hemorrhagic stroke. In terms of enhancing NVU repair, drugs that activate the retinoic acid receptor (RXR) mobilize diverse vascular and glial cell subtypes that suppress inflammation and rebuild the NVU (Bi et al., 2010; Evans and Mangelsdorf, 2014; Pouso and Cairrao, 2022). Remyelination failure that blocks the repair of damaged white matter tracts is another major obstacle in the treatment of stroke (Sozmen et al., 2019). In this regard, TR agonists have been shown to increase functional recovery in animal models of ischemic and hemorrhagic stroke by stimulating the differentiation of oligodendrocyte progenitor cells into myelin-producing oligodendrocytes (Vose et al., 2013; Talhada et al., 2019).

We explain how combining an MCU_*cx*_ inhibitor and NCLX activator with RXR and TR agonists should further improve functional recovery. To maximize the safety and efficacy of this combinatory approach, we describe the use of novel nanoparticle formulations designed to preferentially deliver drugs to brain tissues that are metabolically compromised or have been recently damaged by a stroke.

## 2. Structure and function of the MCU_*cx*_

To appreciate how the MCU_*cx*_ regulates mitochondrial Ca^2+^ uptake, it is necessary to understand the structure and function of the various subunits that comprise the MCU_*cx*_. However, it is not yet clear how these subunits interact to gate the MCU_*cx*_. This problem is complicated by evidence that certain MCU_*cx*_ subunits appear to have broad actions on mitochondrial function. We will therefore only provide a brief overview of this rapidly evolving topic and recommend several excellent recent reviews that provide a comprehensive discussion of the functional architecture of the MCU_*cx*_ (Boyman et al., 2021; Feno et al., 2021b; Garbincius and Elrod, 2022).

### 2.1. Architecture of the MCU_*cx*_

**FIGURE 1:**
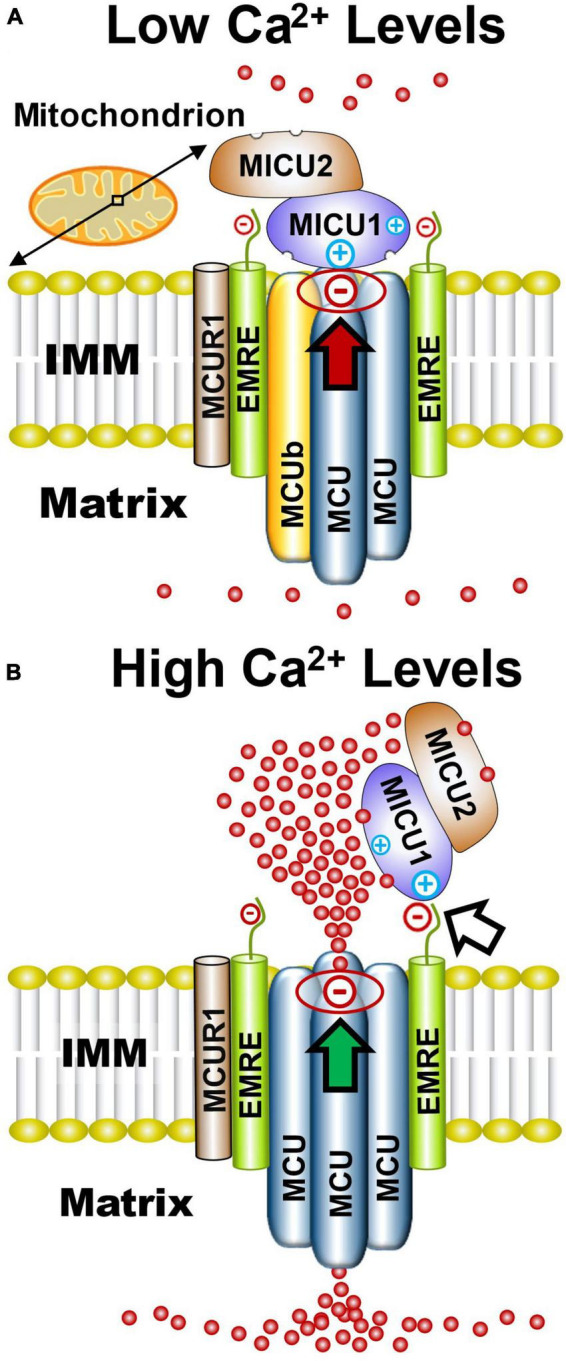
Functional architecture of the MCU_*cx*_ based on structural studies. **(A)** Electrostatic interactions between the channel pore and MICU1 (red arrow) block the MCU complex when Ca^2+^ levels are low. **(B)** Elevated Ca^2+^ levels increase Ca^2+^ binding to MICU1/MICU2 heterodimers that exposes positively charged amino acids in MICU1. This strengthens the association of MICU1 with negatively charged amino acids in EMRE which displaces the MICU1/2 dimer from the channel pore (white arrow) resulting in increased Ca^2+^ influx into the matrix (green arrow). Based on recent cryo-EM studies and size on native gels (∼480 kD), it is likely that the MCU_*cx*_ holocomplex consists of two conjoined dimers of the MCU/EMRE pore which are each associated with a single MICU1/MICU2 heterodimer. (See section “2.1. Architecture of the MCUcx” for abbreviations and mechanistic details).

Since discovery of the MCU subunit that creates the MCU_*cx*_ channel pore (Baughman et al., 2011; Stefani et al., 2011), six accessory subunits have been identified. These include MCUb, mitochondrial Ca^2+^ uptake 1, 2, and 3 (MICU1, MICU2, and MICU3); essential MCU_*cx*_ regulator (EMRE), and MCU regulator 1 (MCUR1) (Figure 1A). Three-dimensional structure analysis of cryogenic microscopy (cryo-EM) images suggests that the MCU creates the channel pore by forming a homo-oligomer of four MCU subunits (Figure 1A; Baradaran et al., 2018; Fan et al., 2018; Nguyen et al., 2018; Yoo et al., 2018). Each MCU subunit has two coiled coil domains and two transmembrane domains separated by a short hydrophilic acid linker composed of a DIME motif (D-X-X-E, where X indicates hydrophobic residues) (Baughman et al., 2011; Stefani et al., 2011; Cao et al., 2017). The carboxylate groups of this DIME motif, strategically located on the pore entrance of the second transmembrane domain of the MCU, appear to mediate the highly selective gating of Ca^2+^ by the MCU_*cx*_ (Oxenoid et al., 2016; Cao et al., 2017; Yoo et al., 2018). MCUb acts an inhibitory subunit that reduces Ca^2+^ conductance by displacing an MCU subunit from the channel pore (Figure 1B; Raffaello et al., 2013; Lambert et al., 2019). EMRE, found only in metazoans, is a protein with a single transmembrane domain (Sancak et al., 2013; Kovacs-Bogdan et al., 2014). In brain, the majority of MCU tetramers appear to associate with two EMREs (Watanabe et al., 2022). Importantly, EMRE is essential for *in vivo* MCU_*cx*_ activity and MCU oligomers alone are not sufficient for *in vivo* uniporter activity (Sancak et al., 2013). MCUR1 acts as a scaffolding protein that facilitates assembly and activity of the MCU_*cx*_ (Tomar et al., 2016). Due to the absence of structural studies, the stoichiometry of the MCUR1 in the MCU_*cx*_ is unknown (Garbincius and Elrod, 2022).

MICU1, MICU2, and MICU3 are similar in size and have a mitochondrial targeting sequence at their amino terminus, and two canonical Ca^2+^-binding EF hands (Csordas et al., 2013; Sancak et al., 2013). MICU1 can form homodimers or heterodimers with MICU2 or MICU3 (Sancak et al., 2013; Wang et al., 2014; Patron et al., 2019). Based on cryo-EM structures of the MCU_*cx*_, it has been theorized that under low Ca^2+^ conditions the MCU_*cx*_ pore is plugged by an MICU1/MICU1, or MICU1/MICU2 dimer (Fan et al., 2020; Wu et al., 2020; Zhuo et al., 2021). Electrostatic interactions between a polybasic sequence (KKKKR) in MICU1 and an aspartate (D261) residue, present in each of the MCU subunits, is considered to block the channel pore (Tsai M. F. et al., 2016; Phillips et al., 2019; Figure 1A). MICU2 lacks these basic residues and therefore requires dimerization with MICU1 to associate with the MCU_*cx*_ (Plovanich et al., 2013; Patron et al., 2014; Payne et al., 2017). EMRE is thought to act as a bridge between the MCU subunits and a homodimer of MICU1 or heterodimer of MICU1 and MICU2 (Sancak et al., 2013; Tsai M. F. et al., 2016). Under high Ca^2+^ conditions, Ca^2+^ binding to MICU1/MICU1, or MICU1/MICU2 dimers induces a confirmational change that strengthens the interaction between a polybasic sequence in MICU1 and the poly-aspartate tail of EMRE (Wang Y. et al., 2019; Figure 1B). This confirmational change is thought to enhance Ca^2+^ entry into the matrix by releasing the dimer from the channel pore (Patron et al., 2014; Payne et al., 2017; Wang Y. et al., 2019; Figure 1B). The lower affinity of MICU2 than MICU1 for Ca^2+^ is considered to enable fine-tuning of MCU_*cx*_ activity by MICU1/MICU2 dimers (Kamer et al., 2017).

The validity of this model has been challenged by direct patch clamp recordings of macroscopic and single MCU_*cx*_ currents in mitoplasts (mitochondria stripped of their outer mitochondrial membrane). In these studies, CRISPR-Cas9 was employed to knockout MICU1 in mouse embryonic fibroblasts (Garg et al., 2021). By comparison to wild-type cells, MICU1 knockout cells showed a reduction in MCU_*cx*_ activity suggesting that MICU1 potentiates rather than suppressing mitochondrial Ca^2+^ uptake (Garg et al., 2021). At odds with this result are studies that indicate familial mutations resulting in loss of MICU1 function (Logan et al., 2014; Bhosale et al., 2017; Wilton et al., 2020; Kohlschmidt et al., 2021) and MICU1 ablation (Mallilankaraman et al., 2012; Liu H. et al., 2016; Singh et al., 2022) render cells more susceptible to mitochondrial Ca^2+^ overloading.

One possibility that may account for these discrepant findings is the reduction of EMRE levels seen in MICU1 knockout cells (Garg et al., 2021; Tsai et al., 2023). Since EMRE depletion inhibits MCU_*cx*_ activity (Sancak et al., 2013; Liu J. C. et al., 2016), suppressed mitochondrial Ca^2+^ uptake in MICU1 knockout cells may reflect a loss of EMRE rather than MICU1. In support of this hypothesis, EMRE rescue restores MCU_*cx*_ activity in MICU1 knockout cells (Tsai et al., 2023). In further support for occlusion of the MCU_*cx*_ pore by MICU1, ion permeation through this uniporter is suppressed by MICU1 under divalent-free conditions (Rodríguez-Prados et al., 2023).

### 2.2. MICU1 regulates cristae junction dynamics and spatially anchors the MCU_*cx*_

**FIGURE 2:**
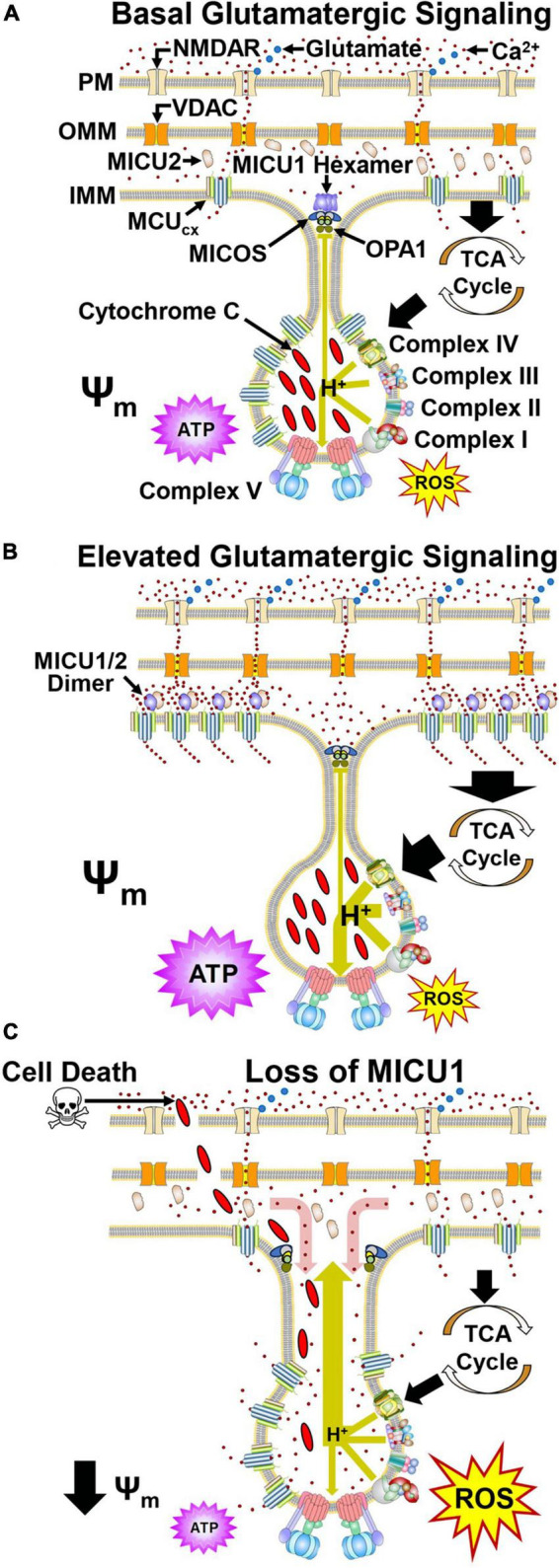
Dynamic regulation of mitochondrial Ca^2+^ handling and respiration by MICU1 and OPA1 in cristae. **(A)** Glutamate (blue) activates the NMDA receptor (NMDAR) resulting in Ca^2+^ influx. Ca^2+^ then crosses the OMM via VDAC. Under basal conditions, MCU and EMRE subunits confer MCU_*cx*_ activity that transports Ca^2+^ into the matrix. Ca^2+^ allosterically activates dehydrogenases in the tricarboxylic acid cycle (TCA) that generate reducing equivalents which drive proton (H^+^) pumping by Complexes I, III, and IV (large green arrow). ATP synthase harvests this proton gradient to manufacture ATP. MICOS, OPA1, and MICU1 hexamers located at the cristae junction (CJ) oppose the movements of Ca^2+^ into and H^+^ out (small green line) of the cristae lumen. **(B)** Increased glutamate release enhances NMDAR activation resulting in elevated Ca^2+^ entry into the cytosol. VDAC then transports greater amounts of Ca^2+^ across the OMM. The resultant elevation of Ca^2+^ in the intramembrane space triggers the dissociation of MICU1 subunits that recruit MCU and EMRE subunits from the cristae. **(C)** Pathological depletion of MICU1 opens the CJ allowing Ca^2+^ to enter (red arrow) and cytochrome c and H^+^ to escape (large green arrow) the cristae lumen. This uncouples the electron transport chain resulting in reduced ATP synthesis, increased ROS production and cytochrome c-induced cell death. Adapted from Gottschalk et al., 2022. (See section “2.2. MICU1 regulates cristae junction dynamics and spatially anchors the MCUcx” for abbreviations and mechanistic details).

The inner mitochondrial membrane (IMM) is composed of two compartments known as cristae and the inner boundary membrane (IBM) (Palade, 1953). Cristae are folds that protrude into the matrix while the IBM extends parallel to the length of the outer mitochondrial membrane (OMM) (Perkins et al., 1997). Narrow tubular-like structures, called crista junctions (CJs), connect cristae with the IBM (Perkins et al., 1997). Complexes I-IV of the electron respiratory chain are positioned along the lateral walls of cristae (Vogel et al., 2006; Wilkens et al., 2013) while dimers of ATP synthase (Complex V) are arranged in rows at the edge of cristae (Dudkina et al., 2005; Davies et al., 2011; Figure 2A). CJs are kept in a closed state by oligomers of the inner-membrane dynamin-like GTPase, OPA1 (Frezza et al., 2006) and the mitochondrial contact site and cristae organizing system (MICOS complex) (John et al., 2005; Rabl et al., 2009; Barrera et al., 2016). This arrangement enables each crista to function as an independent bioenergetic unit that prevents the failure of one from propagating dysfunction to others (Wolf et al., 2019).

Depolarization of neurons by the activation of ionotropic glutamatergic receptors (N-methyl-D-aspartate, NMDA) on the cell surface triggers Ca^2+^ influx into the cytosol (Hansen et al., 2021; Figure 2A). Ca^2+^ then enters the mitochondrial intramembrane space (IMS) via the voltage-dependent anion-selective channel (VDAC) (Shoshan-Barmatz et al., 2010). However, until recently, how MCU, EMRE, and MICU1 spatially interact to regulate the subsequent rise in mitochondrial Ca^2+^ uptake and energy production has been unclear. In this respect, the combined use of super-resolution structured illumination microscopy, electron microscopy and sub-mitochondrial Ca^2+^ recordings have yielded important insights. These techniques have permitted changes in the mitochondrial localization of MCU, EMRE, and MICU1 to be monitored under basal and elevated Ca^2+^ conditions in living cells. Studies that have employed them indicate during resting conditions, MICU1 exists as a hexamer which stabilizes the CJ by interactions with OPA1 and MICOS (Gottschalk et al., 2019; Tomar et al., 2019; Figure 2A). Under resting conditions, MCU and EMRE subunits, located throughout the IMM, mediate Ca^2+^ entry into the matrix (Gottschalk et al., 2019, 2022). With a physiological elevation of cytosolic Ca^2+^ concentrations, the subsequent rise of IMS Ca^2+^ levels triggers dissociation of the MICU1 hexamer resulting in opening of the CJ (Gottschalk et al., 2019, 2022; Figure 2B). MICU1 then forms homodimers or heterodimers with MICU2 that recruit MCU and EMRE subunits from the cristae to the IBM that regulate MCU_*cx*_ activity (Gottschalk et al., 2019, 2022).

In *Drosophila*, a loss-of-function mutation in the MICU1 caused lethality that was not mitigated by a loss-of-function mutation in the MCU which blocks mitochondrial Ca^2+^ uptake (Tufi et al., 2019). Furthermore, like OPA1 deletion and mutations that impair MICOS assembly (Olichon et al., 2003; Guarani et al., 2015; Zhou et al., 2018), MICU1 knockdown alters cristae morphology (Gottschalk et al., 2019, 2022). These findings, indicating that MICU1 influences cell survival by a non-MCU_*cx*_ mechanism, led to the identification of MCU_*cx*_-independent MICU1 interactors. Coimmunoprecipitation assays using wild-type MICU1 and various MICU1 mutants demonstared that the C terminal domain of MICU1 directly interacts with the MICOS components MIC60 and CHCHD2 in an MCU_*cx*_-independent manner (Tomar et al., 2023). Measurements of mitotchondrial structure in MICU1 null cells revealed an increase in both the inter-CJ distance and CJ width relative to wild-type cells (Tomar et al., 2023). As a result, the regular spacing of the mitochondrial membrane potential (Ψ_*m*_) in peaks along the cristae was lost. This suggests that disruption of the CJ in MICU1 knockout cells allows protons to escape resulting in depolarization of the Ψ_*m*_ (Figure 2C). Opening of the CJ was then shown to promote cell death by enabling release of the pro-apoptotic factor cytochtome c from the cristae (Tomar et al., 2023; Figure 2C). MCU deletion in MICU1 knockout cells did not suppress cytochrome c release triggered by the pro-apoptotic protein tBid (Tomar et al., 2023). This suggests that MICU1 deletion does not promote cell death by increasing mitochondrial Ca^2+^ uptake. Depolarization of the Ψ_*m*_ disrupts electron transport resulting in energy failure and excessive ROS production suggesting that these mechanisms may contribute to cell death by MICU1 loss (Murphy, 2009; Figure 2C). Assuming that Ca^2+^ concentrations are lower in cristae than the IMS, CJ opening in cells lacking MICU1 may raise Ca^2+^ levels in crisate (Figure 2C; Gottschalk et al., 2019). However, to determine if this is the case and whether Ca^2+^ concentrations in the IMS regulate mitochondrial ultrastructure by modulating cristae organization (Gottschalk et al., 2018, 2019), the development specific Ca^2+^ sensors for the MICOS complex or CJ are required (Tomar et al., 2023).

### 2.3. The MICU3 subunit potently elevates mitochondrial Ca^2+^ uptake

MICU3 is expressed predominantly in brain and skeletal muscle (Kovacs-Bogdan et al., 2014; Oxenoid et al., 2016). MICU3 acts as a highly potent stimulator of MCU_*cx*_ activity (Patron et al., 2019). The greater affinity of MICU3 than MICU2 for Ca^2+^ enables MICU1/3 dimers to become activated at lower cytosolic Ca^2+^ concentrations than MICU1/MICU1, or MICU1/2 dimers (Patron et al., 2014, 2019; Kamer et al., 2017). This supports the high metabolic needs of neurons by allowing small and fast increases of cytosolic Ca^2+^ concentrations associated with enhanced glutamatergic signaling to increase MCU_*cx*_ activity (Patron et al., 2019; Ashrafi et al., 2020). However, the ability of MICU3 to lower the Ca^2+^ threshold for MCU_*cx*_ activation comes at a price by increasing the risk of mitochondrial Ca^2+^ overloading. This is supported by evidence that MICU3 knockdown protects the brain from damage in a rat model of hemorrhagic stroke (Wang et al., 2023) and MICU3 deletion reduces ischemic/reperfusion injury in the heart (Puente et al., 2020). Since MICU3 increases mitochondrial Ca^2+^ uptake by dimerizing with MICU1 (Patron et al., 2019), drugs that reversibly disrupt the formation of MICU1/3 dimers may be a useful protective strategy for stroke.

## 3. Impact of MCU_*cx*_ subunit mutations on the human brain

### 3.1. Loss-of-function MICU1 mutations produce abnormal involuntary movements indicative of manganese neurotoxicity

Loss-of-function mutations in MICU1 have been linked to a muscle and brain disorder characterized by muscle weakness, cognitive deficits, and abnormal involuntary movements including chorea, tremor, dystonic posturing, and orofacial dyskinesias (Logan et al., 2014; Bhosale et al., 2017; Mojbafan et al., 2020; Wilton et al., 2020; Kohlschmidt et al., 2021). Studies performed using fibroblast and lymphoblasts derived from these patients indicate that MICU1 deficiency causes a chronic activation of the MCU_*cx*_, even in the presence of low cytosolic Ca^2+^ concentrations, resulting in mitochondrial damage (Logan et al., 2014; Bhosale et al., 2017; Wilton et al., 2020; Kohlschmidt et al., 2021). The recent generation of neuronal MICU1 knockout mice has confirmed that impaired MICU1 function causes neurodegeneration (Singh et al., 2022). Primary cultures of cortical neurons derived from these mice display greater susceptibility to mitochondrial Ca^2+^ overloading, mitochondrial permeability transition pore opening, and excitotoxic cell death relative to wild-type neurons (Singh et al., 2022). Like MICU1-deficient patients, neuronal MICU1 deficient mice develop a progressive loss of motor and cognitive function associated with the degeneration of motor neurons in the spinal cord and cortex (Singh et al., 2022).

The abnormal involuntary movements produced by familial MICU1 mutations are remarkably similar to those observed in individuals suffering from manganese (Mn^2+^) toxicity (Parmalee and Aschner, 2016). The globus pallidus is particularly sensitive to the neurotoxic effects of Mn^2+^ (Pal et al., 1999). Magnetic resonance imaging (MRI) has revealed signs of damage in the globus pallidus of individuals with loss-of-function MICU1 mutations (Logan et al., 2014). In keeping with these observations, MICU1 ablation sensitizes human cells to Mn^2+^-induced cell death by increasing mitochondria uptake of this heavy metal (Kamer et al., 2018; Wettmarshausen et al., 2018). These findings suggest that mitochondrial overloading with Mn^2+^, rather than Ca^2+^, is responsible for the clinical manifestations of MICU1 mutations. Interestingly, Mn^2+^ accumulation in the brain has also been implicated in Parkinson’s disease, stroke, and Alzheimer’s disease (Li and Zhang, 2012; Martins et al., 2019) suggesting that MCU_*cx*_ inhibitors maybe useful in the treatment of a broad range of neurodegenerative disorders.

### 3.2. Mutation of MICU2 causes cognitive deficits and disrupts mitochondrial function

A homozygous mutation that truncates MICU2 has been reported to fully segregate with a neurodevelopmental disorder in a multiplex consanguineous family (Shamseldin et al., 2017). Unlike MICU1 deficiency, MICU2 truncation produces cognitive deficits without motor involvement (Shamseldin et al., 2017). MRI has revealed bilateral gliosis in parietal periventricular regions and multiple T2 hyperintensity foci scattered within both cerebral hemispheres with a subcortical distribution (Shamseldin et al., 2017). Consistent with intact motor function, no signs of basal ganglia damaged were reported (Shamseldin et al., 2017). Skin fibroblasts derived from MICU1- or MICU2-deficient patients display cytoplasmic Ca^2+^ overloading during resting states. However, unlike MICU1 deficiency, MICU2 loss slows mitochondrial Ca^2+^ influx (Shamseldin et al., 2017). This difference may reflect a compensatory increase in NCLX activity that enhances extrusion of Ca^2+^ from the mitochondrial matrix of MICU2 deficient cells (Palty et al., 2010; Bhosale et al., 2017). In further contrast to the effects of MICU1 deficiency, MICU2 ablation elevates the ΔΨ_*m*_ (Shamseldin et al., 2017). This maybe due to NCLX activation that increases mitochondrial sodium (Na^+^) concentrations. The subsequent induction of Na^+^/hydrogen exchange by elevated Na^+^ concentrations in matrix would thus increase the ΔΨ_*m*_ by enhancing proton pumping into the IMS (Bhosale et al., 2017). The reason(s) for these differences between the effects of MICU1- and MICU2-deficiency are unclear but may reflect the impact of variations in the tissue distributions of these subunits (Plovanich et al., 2013).

### 3.3. Mitochondrial-AAA protease mutations activate the MCU_*cx*_ by blocking the proteolytic degradation of EMRE

Mutations in subunits of mitochondrial ATPases associated with diverse cellular activities (mitochondrial-AAA) proteases cause neurodegeneration in spinocerebellar ataxia type 28, hereditary spastic paraplegia 7, and spastic ataxia 5 (Casari et al., 1998; Di et al., 2010; Pierson et al., 2011). Heterozygous deletion of the Afg3l2 subunit of the mitochondrial-AAA protease in mice produces a progressive loss of motor coordination accompanied by the death of Purkinje neurons in the cerebellar cortex. In these neurons, mitochondria appear swollen and display disrupted cristae suggestive of mitochondrial Ca^2+^ overloading (Maltecca et al., 2009, 2015). Loss of the mitochondrial-AAA protease elevates mitochondrial Ca^2+^ uptake that partially mimics the disruption of MCU_*cx*_ gatekeeping caused by downregulation of the MICU1 (Konig et al., 2016). This occurs because in the absence of the mitochondrial-AAA protease, non-assembled EMRE subunits are no longer degraded resulting in the formation of constitutively active EMRE-MCU channels (Konig et al., 2016). These findings account for the similar clinical features produced by MICU1 and mitochondrial-AAA protease deficiencies.

### 3.4. Transmembrane BAX Inhibitor Motif containing protein 5 (TMBIM5) is a mitochondrial Ca^2+^/H^+^ exchanger and inhibitor of the mitochondrial-AAA protease

TMBIM5 is a mitochondrial Ca^2+^/H^+^ uniporter that utilizes the high proton gradient generated by the Ψ_*m*_ to power Ca^2+^/H^+^ exchange (Austin et al., 2022; Patron et al., 2022). This mitochondrial Ca^2+^/H^+^ exchanger maintains cell survival and respiration by stimulating mitochondrial Ca^2+^ efflux and limiting hyperpolarization of the Ψ_*m*_ (Patron et al., 2022). Under normal conditions, TMBIM5 blocks mitochondrial-AAA protease activity (Patron et al., 2022). This maintains respiration by preventing the premature degradation of unassembled or damaged respiratory chain subunits. However, persistent hyperpolarization of the Ψ_*m*_ renders TMBIM5 susceptible to proteolysis by mitochondrial-AAA protease. The subsequent enhancement of mitochondrial-AAA protease activity results in the degradation of multiple Complex I subunits to limit injurious ROS over-production in hyperpolarized mitochondria (Patron et al., 2022). The physiological importance of TMBIM5 has been demonstrated by increased embryonic or perinatal mortality and skeletal myopathy in mice lacking a region critical for the activity of this Ca^2+^/H^+^ exchange (Zhang et al., 2022).

## 4. Contrasting effects of global and conditional MCU ablation on resistance to ischemic/reperfusion injury

### 4.1. Global MCU ablation fails to protect the heart from ischemic/reperfusion injury

Identification of the MCU has made it possible to study the effects of MCU ablation in mice. In keeping with a critical physiological role of the MCU, global deletion of the MCU is lethal in C57/BL6 mice at E11.5-13.5, possibly because of cardiac failure (Pan et al., 2013; Murphy et al., 2014). By crossing different strains of mice to obtain offspring with a mixed genetic background it has been possible to generate MCU nulls (Pan et al., 2013). As expected, mitochondrial Ca^2+^ uptake is markedly suppressed in skeletal muscle derived from MCU nulls (Pan et al., 2013). However, global MCU ablation does not protect the heart from ischemic/reperfusion injury considered to be mediated by excessive mitochondrial Ca^2+^ uptake (Pan et al., 2013).

### 4.2. Global MCU ablation causes a switch from oxidative phosphorylation to glycolysis that lowers resistance to ischemic/reperfusion brain injury

We have also shown that mitochondrial Ca^2+^ uptake is suppressed in mitochondria isolated from the brains of MCU nulls (Nichols et al., 2017). However, global MCU ablation failed to preserve mitochondrial function in cortical neuron cultures subjected to a lethal period of oxygen glucose deprivation (OGD), an *in vitro* model of ischemic/reperfusion brain damage (Nichols et al., 2017). In the case of hypoxic/ischemic brain damage, we also found that wild-type littermates and MCU null mice displayed comparable sensorimotor deficits, mitochondrial injury in CA1 hippocampal neurons and neuronal damage in the striatum, hippocampus, and cortex (Nichols et al., 2017). Interestingly, global MCU ablation blocked the protective effects of hypoxic preconditioning. Unlike wild-type mice, hypoxic preconditioning failed to reduce sensorimotor deficits and neuronal damage in MCU nulls subjected to hypoxic/ischemic brain injury (Nichols et al., 2017). Examination of mitochondrial function and glycolysis in respiring cortical neuron cultures derived from wild-type and MCU null mice revealed a switch from oxidative phosphorylation to glycolysis for energy production in MCU nulls (Nichols et al., 2017). Relative to wild-type cortical neurons, energetic stress enhanced glycolysis and depressed Complex I activity in MCU null cortical neurons. Following transient hypoxia/ischemia, forebrain levels of the reduced form of nicotinamide adenine dinucleotide (NADH) were decreased more in MCU nulls than wild-type mice suggesting that increased glycolytic consumption of NADH suppressed Complex I activity. Compared to wild-type cortical neurons, pyruvate dehydrogenase was hyper-phosphorylated in MCU null neurons at several sites that lower the supply of substrates for the tricarboxylic acid cycle. However, the elevation of cytosolic Ca^2+^ with glutamate or ionomycin decreased pyruvate dehydrogenase phosphorylation in MCU null neurons suggesting the use of alternative mitochondrial Ca^2+^ transport. Relative to wild-type mice subjected to hypoxic preconditioning, untreated MCU nulls showed similar increases of Ca^2+^ handling genes in the hippocampus. Alternative Ca^2+^ handling mechanisms that compensate for lost mitochondrial Ca^2+^ uptake may thus compromise resistance to hypoxic/ischemic brain injury and disrupt hypoxic preconditioning in MCU nulls (Nichols et al., 2017).

### 4.3. Conditional MCU ablation at adulthood reduces ischemic/reperfusion damage in the heart and brain

Unlike constitutive (global) MCU ablation, inducible MCU deletion in cardiac myocytes at maturity protected the heart from ischemic/reperfusion injury (Kwong et al., 2015; Luongo et al., 2015). These differences may be explained by upregulation of the receptor-interacting protein 3 kinase death pathway in global MCU nulls (Parks et al., 2019). To examine if the MCU is also involved in hypoxic/ischemic brain injury, we generated conditional knockouts in which the MCU was selectively deleted in Thy1-expressing neurons at maturity. Relative to Thy1 genetic controls, hypoxic/ischemic-induced sensorimotor deficits, forebrain neuron loss and mitochondrial damage were decreased in these Thy1-MCU deficient mice (Nichols et al., 2018). MCU knockdown by siRNA-induced silencing in cortical neuron cultures also reduced cell death and mitochondrial respiratory deficits by a lethal period of OGD (Nichols et al., 2018). Importantly, MCU silencing did not produce metabolic abnormalities observed previously in cortical neuron cultures derived from global MCU nulls (Nichols et al., 2017, 2018). These findings indicate that metabolic adaptations that occur during neurodevelopment render global MCU nulls susceptible to hypoxic/ischemic brain injury. Furthermore, they suggest that MCU inhibitors are most likely to have greatest therapeutic benefit for the acute management of ischemic/reperfusion brain injury.

## 5. Structure and function of the sodium/Ca^2+^/lithium exchanger (NCLX)

We have shown that the NCLX mediates the extrusion of Ca^2+^ from mitochondria (Palty et al., 2010) and identified drugs that oppose injurious mitochondrial Ca^2+^ overloading in neurons by increasing NCLX activity (Rozenfeld et al., 2022). To appreciate how the NCLX can be targeted to enhance mitochondrial Ca^2+^ efflux, it is necessary to describe the structural and functional features of this Na^+^/Ca^2+^ exchanger.

### 5.1. Functional architecture of the NCLX

**FIGURE 3:**
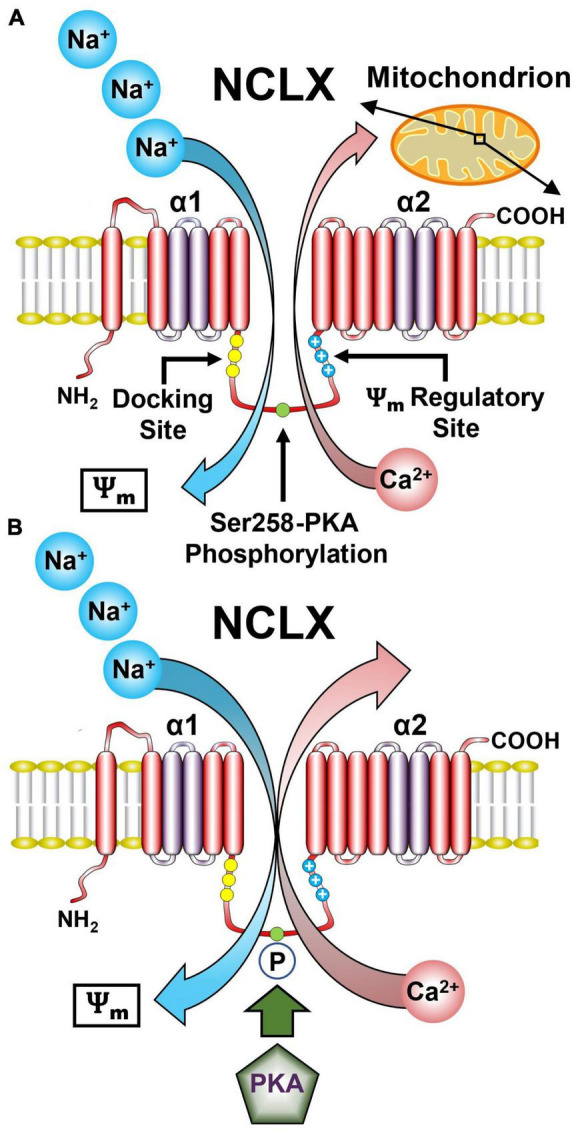
Representative model of the NCLX showing the regulatory domains and sites. **(A)** The NCLX regulatory loop is located between the two catalytic α1 and α2 domains. NCLX has a Ψ_*m*_ sensing allosteric site as well as a PKA-dependent phosphorylation site Ser258. **(B)** The Ψ_*m*_ provides the driving force for the electrogenic transport of three Na^+^ ions for one Ca^2+^ ion. Phosphorylation of Ser258 by PKA increases NCLX activity. (See section “5.1. Functional architecture of the NCLX” for abbreviations and mechanistic details).

The mitochondrion is not only the energy center but also the Ca^2+^ signaling hub of the cell. As described in the previous sections, Ca^2+^ flows into the mitochondria via the MCU_*cx*_. Ca^2+^ is then pumped out mostly by a Na^+^/Ca^2+^ exchanger (Stefani et al., 2016). The activity of the mitochondrial Na^+^/Ca^2+^ exchanger was discovered in 1974 (Carafoli et al., 1974), but its molecular identity was not resolved until 2010 and found to be linked to the NCLX gene, a member of the Na^+^/Ca^2+^ exchanger (NCX) superfamily (Palty et al., 2010). Phylogenetic analysis shows that NCLX is a single member of a distinct and early branch in the NCX superfamily (Lytton, 2007). The 3-D structure of NCLX is unresolved. However molecular modeling of the NCLX fitted to the structure of the bacterial NCX homologue (NCX-MJ) shows striking conservation of the catalytic site and overlap of the Na^+^ and Ca^2+^ transport sites in the α1 and α2 domains (Figure 3A). Nevertheless, there are important functional differences between these domains for the NCLX and the rest of the NCX family. Most notably, NCX members effectively discriminate between Na^+^ and Li^+^ but NCLX transports Li^+^ and Na^+^ in exchange for Ca^2+^ (Khananshvili, 2017). The latter property was essential for the molecular identification of NCLX. Moreover, recent studies have mapped the residues of NCLX that facilitates Li^+^ transport (Roy et al., 2017).

Another uncertainty regarding the NCLX is the stoichiometry of Na^+^ and Ca^2+^ transported by this exchanger. NCX members mediate the electrogenic exchange of 3–4 Na^+^ for 1 Ca^2+^ and electrophysiological as well as kinetic studies have suggested that NCLX shares the same cation stoichiometry (Islam et al., 2020; Figure 3A). However, recent studies based on NCLX NCX-MJ chimeras suggest an electroneutral 2 Na^+^/Ca^2+^ exchange ratio (Giladi et al., 2022). This difference in stoichiometry has important physiological implications because it determines the mitochondrial Na^+^ and Ca^2+^ gradient driven by NCLX. Further studies are required to resolve this issue.

Another important difference between NCX and NCLX is the structure and function of the regulatory loop joining the α1 and α2 catalytic domains. The regulatory loop of NCX, particularly NCX1 harbors an allosteric Ca^2+^ binding site (Khananshvili, 2017). Instead, the NCLX regulatory loop contains several phosphorylation sites notably protein kinase A (PKA) and Ca^2+^/calmodulin-dependent kinase 2 sites that control NCLX activity (Katoshevski et al., 2022). The PKA site is also controlled by other players most notably the mitochondrial phosphodiesterase 2 (PDE2) that by controlling mitochondrial cyclic adenosine monophosphate (cAMP) levels regulates NCLX activity through its PKA site (Rozenfeld et al., 2022; Figure 3B). In addition, NCLX regulatory domain contains a cluster of positively charged residues that may form a membrane crossing helix which might enter the membrane and looks strikingly like a channel voltage sensor (Kostic et al., 2018). Studies monitoring NCLX activity under varying mitochondrial membrane potentials suggest that this site senses the Ψ_*m*_ and accordingly tunes NCLX activity.

## 6. Pharmacological strategies to protect and repair the NVU

**FIGURE 4:**
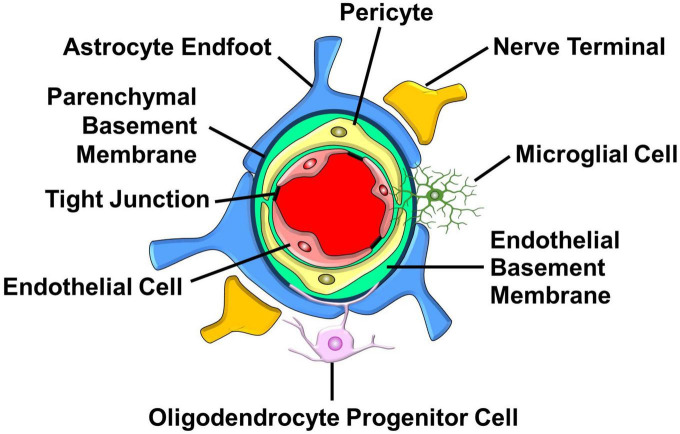
Major structural and cellular components of the capillary NVU. Endothelial cells that line the capillary lumen form tight junctions which oppose the movement metabolites and proteins into the brain. Pericytes located in the endothelial basement membrane control capillary diameter. Astrocyte endfeet that surround the parenchymal basement sense increases in neuronal activity and respond by releasing vasoactive factors. Nerve terminals, found in proximity with astrocyte endfeet, release neurotransmitters that influence capillary diameter. Oligodendrocyte progenitor cells interact with cellular components of the NVU to regulate the formation and function of capillaries. Microglia send processes into the NVU that allow them to communicate with pericytes and endothelial cells. (See section “6.1. Structure and function of the NVU” for details).

The NVU is an intricate structure comprised of vascular cells (smooth muscle cells, endothelial cells, and pericytes), glia (astrocytes, oligodendrocytes, and microglia), and neurons that communicate with each other to regulate CBF (Iadecola, 2004; Kugler et al., 2021). As cerebral blood vessels plunge deeper into the brain to become capillaries, smooth muscle cells are lost while pericytes are gained (Armulik et al., 2010; Sweeney et al., 2016; Figure 4). Computational analysis of cerebral perfusion and oxygen supply suggests that the capillary bed is the largest contributor to hydraulic resistance in the brain (Gould et al., 2017). The fine tuning of capillary diameter therefore enables precise local control of CBF (Hartmann et al., 2021). The bulk of metabolite and gas exchange between brain cells and the blood also occurs in the capillary bed (Jespersen and Østergaard, 2012). Furthermore, capillaries are a major source of neurotrophic signals that maintain neuronal health (Nikolakopoulou et al., 2019). Lastly, capillaries sense elevated extracellular potassium levels associated with increased neuronal activity and respond by sending a retrograde signal that dilates arterioles resulting in enhanced CBF (Longden et al., 2017). However, the delicate cytoarchitecture of capillaries is easily damaged by a stroke but not so easily repaired, especially in the elderly (Chen et al., 2010; Iadecola, 2013; Mastorakos et al., 2021). For these reasons, we will focus on the structure and function of capillary NVUs and the importance of mitochondrial fidelity for NVU function and integrity. We will then discuss the potential advantages of targeting the MCU_*cx*_ and NCLX to protect the NVU, and RXR activation to repair the NVU after a hemorrhagic or ischemic stroke. For a comprehensive description of the structure and function of the NVU in the healthy brain and the deleterious effects of a stroke on NVU integrity and function, we recommend several outstanding reviews of these complex topics (Iadecola, 2017; McConnell et al., 2017; Kaplan et al., 2020).

### 6.1. Structure and function of the NVU

Endothelial cells form the wall of cerebral blood vessels (Iadecola, 2004). Tight junctions between endothelial cells create a physical barrier that impedes the paracellular diffusion of ions, macromolecules, and other polar solutes (Stamatovic et al., 2008). Endothelial cells also act as a biochemical barrier by selectively transporting nutrients into the brain from the blood and exporting solutes and metabolite waste products from the brain into the blood. This is achieved by transporters, metabolite-degrading enzymes, receptors, ion channels, and ion transporters situated on the luminal and/or abluminal membranes of endothelial cells (Loscher and Potschka, 2005; Iadecola, 2017). Endothelial cells also secrete a rich repertoire of trophic factors that sustain and repair the NVU (Shen et al., 2004; Goldman and Chen, 2011). These dynamic cells play a key role in neurovascular coupling, a phenomenon in which increased neuronal activity enhances CBF (Iadecola, 2017). Increased neuronal activity elevates extracellular potassium concentrations that are sensed by endothelial cells. The increased entry of potassium via capillary endothelial cell inward-rectifier potassium (KIR2.1) channels produces a rapidly propagating retrograde hyperpolarization that causes upstream arteriolar dilation thus increasing blood flow into the capillary bed (Longden et al., 2017).

Pericytes are located within the endothelial basement membrane that surrounds blood vessels (Winkler et al., 2011). This positions pericytes between endothelial cells, astrocytes, and neurons thus allowing them to receive signals from these adjacent cells and mount responses essential for proper NVU function (Sweeney et al., 2016). Pericytes have diverse roles that include vessel maintenance and permeability, angiogenesis, clearance of cellular debris, immune cell entry, and CBF regulation (Daneman et al., 2010b; Attwell et al., 2016; Sweeney et al., 2016). These mural cells are also critical for formation of the NVU by inducing the polarization of astroglial endfeet around cerebral blood vessels (Armulik et al., 2010). Pericytes have also been reported to adopt a stem cell-like phenotype that may enable them to differentiate into vascular and neural cells which might reconstruct NVUs damaged by a stroke (Nakagomi et al., 2015; Nakata et al., 2017; Sun et al., 2020).

Astrocytes are situated between endothelial cells and neurons (Abbott et al., 2006). This strategic location allows them to rapidly adjust CBF in response to changes in synaptic activity and neuronal metabolism (Gordon et al., 2007; Petzold and Murthy, 2011; Muoio et al., 2014). Astrocytes extend endfoot processes that cover the entire abluminal surface of cerebral blood vessels (Mathiisen et al., 2010). These processes display high levels of aquaporin-4, a water channel that is thought to enable waste clearance by the glymphatic system (Díaz-Castro et al., 2023). During brain development, astrocyte endfeet release growth factors that induce the formation of tight junctions and increase the production of transporter proteins in endothelial cells (Alvarez et al., 2013). This establishes bidirectional signaling between astrocyte endfeet and endothelial cells necessary for the regulation of cerebral vascular function and maintenance of NVU integrity (Gordon et al., 2007; Petzold and Murthy, 2011; Muoio et al., 2014).

Nerve terminals in the cerebral cortex, derived from locus coeruleus axons, are positioned very close to (about 1.3 μm) capillaries (Paspalas and Papadopoulos, 1996; Cohen et al., 1997). Norepinephrine release from these axons produces a tonic increase in vascular resistance by stimulating the contraction of pericytes (Giorgi et al., 2020; Korte et al., 2023). This allows elevated neuronal activity to increase CBF by relaxing pericytes (Giorgi et al., 2020; Korte et al., 2023). Interneurons play a key role in neurovascular coupling. They extend processes that terminate beside astrocytes and release vasoactive substances such as vasoactive intestinal peptide and nitric oxide that increase vascular diameter and somatostatin that reduces vascular diameter (Cauli et al., 2004). Optogenetic studies have shown that activation of these interneurons increases local CBF (Anenberg et al., 2015; Krogsgaard et al., 2023). Within the hippocampus, pericyte-covered capillaries are situated near pyramidal neurons (about 8 μm) (Lovick et al., 1999) and are therefore close enough to readily receive signals directly from these neurons (Lecrux and Hamel, 2011). Indeed, optogenetic studies have demonstrated excitatory neuron activation increases local blood flow (Lee et al., 2010; Mester et al., 2019). Pyramidal neurons promote neurovascular coupling by releasing prostaglandins that relax capillaries by activating prostaglandin EP2 and EP4 receptors located on pericytes (Lacroix et al., 2015). However, it is also possible that glutamate release from excitatory neurons may contribute to neurovascular coupling by activating ionotropic and metabotropic glutamate receptors located on astrocytes. In this case, the resultant elevation of intracellular Ca^2+^ concentrations are thought to activate Ca^2+^-sensitive phospholipase A2 resulting in the increased production and release of prostaglandins that dilate the cerebrovasculature by acting on vascular smooth muscle cells and pericytes (Haydon and Carmignoto, 2006; MacVicar and Newman, 2015; Mishra et al., 2016). Neurovascular coupling is therefore mediated by a complex interplay between different neuronal subtypes with endothelial cells, pericytes, and astrocytes (Iadecola, 2017).

Oligodendrocyte progenitor cells (OPCs) differentiate into myelin-producing oligodendrocytes essential for functional recovery after a demyelinating injury (Franklin and Ffrench-Constant, 2008). However, it is now clear that OPCs share close physical and signaling interactions with brain capillaries that strongly influence myelination and angiogenesis (Kisler et al., 2021). As the brain matures, OPCs use the immature vasculature as a platform, allowing them to move along and jump between blood vessels. Genetic ablation of the G-coupled adhesion receptor ADGRA2/Gpr124, necessary for vascular sprouting, blocks OPC migration resulting in a buildup of OPCs within the ventral spinal cord and brain (Tsai H. H. et al., 2016). OPC migration is mediated by the chemokine receptor CXCR4 on OPCs and the ligand for this receptor CxCl12 is secreted by endothelial cells (Tsai H. H. et al., 2016). The movement of OPCs along blood vessels is also supported by the release of vascular endothelial growth factor from endothelial cells (Hayakawa et al., 2011). The development of astrocyte endfeet promotes the detachment OPCs from the cerebrovasculature resulting in their differentiation into oligodendrocytes (Su et al., 2023). Hypoxia in OPCs blocks their differentiation thus providing a mechanism to ensure that sufficient vascularization occurs to meet the high metabolic demands of myelination (Yuen et al., 2014). Finally, OPCs also support development of the cerebrovasculature. Genetic depletion of OPCs severely reduces vascular ramifications and connections in the cortex (Minocha et al., 2015). These findings elegantly demonstrate the importance of reciprocal interactions between OPCs and the cerebrovasculature for the development and integrity of the NVU.

Microglia are resident macrophages in the brain (Ransohoff and Perry, 2009). Approximately one third of all microglial are capillary-associated microglia in the adult mouse brain (Bisht et al., 2021). During development in the mouse and human brain, microglia migrate along the vasculature and become stationary at adulthood in the areas of large capillaries lacking astrocyte endfeet (Mondo et al., 2020). Unlike perivascular microglia that are situated within the parenchymal basement membrane, capillary-associated microglia are located outside of the parenchymal basement membrane (Haruwaka et al., 2019; Mondo et al., 2020; Bisht et al., 2021). However, microglia also extend processes that contact capillary pericytes, endothelial cells, and nerve terminals (Császár et al., 2022). This close association with endothelial cells is thought to allow microglia to maintain BBB integrity by supplying tight-junction proteins (Halder and Milner, 2019; Haruwaka et al., 2019). The purinergic P2Y12 receptor on the cell surface of microglia enables them to participate in neurovascular coupling. Enhanced neuronal activity triggers the release of ATP from nerve terminals that activates microglial P2Y12 receptors (Eyo et al., 2014; Milior et al., 2020). Microglia then respond by releasing vasoactive substances and inflammatory mediators, potentially nitric oxide, prostaglandins, IL-1β, TNF-α, or ROS (Ransohoff and Perry, 2009), that influence the activity of pericytes and endothelial cells (Császár et al., 2022). In keeping with an important role for microglia in neurovascular coupling, genetic ablation of the P2Y12 receptor or microglial depletion impairs vasodilative responses to increased brain activity (Bisht et al., 2021; Császár et al., 2022). Microglial activation after a stroke damages the NVU by their excessive release of pro-inflammatory mediators (Ronaldson and Davis, 2020). Conversely, the polarization of microglia from a pro-inflammatory M1 to an anti-inflammatory M2 phenotype resolves inflammation, protects the NVU, and promotes the differentiation of progenitor cells into new neural and vascular cells which reconstruct the NVU (Ronaldson and Davis, 2020).

### 6.2. Mitochondrial fidelity is crucial for NVU function and integrity

Mitochondria play a critical role in supporting NVU function and integrity by buffering cytosolic Ca^2+^, producing ATP, and synthesizing lipids in vast amounts (Reichard and Asosingh, 2019; An et al., 2021). Myelin synthesis is one of the most energetically expensive processes in the brain that requires mitochondrial biogenesis to produce massive amounts of energy and fatty acids (Harris and Attwell, 2012). Inhibition of Complex I activity with a very low concentration of rotenone (1 nM) that does not reduce OPC viability completely blocks OPC differentiation (Schoenfeld et al., 2010). Genetic ablation of the Complex I subunit NDUFS2 (Cabello-Rivera et al., 2019) and mitochondrial transcription factor A (Beckervordersandforth et al., 2017) also inhibit OPC differentiation. Indeed, myelination deficits and oligodendrocyte loss are common features of mutations that cause mitochondrial dysfunction in humans (Ohara et al., 1988; Zuchner et al., 2004; Kovacs et al., 2005; Lax et al., 2012). In addition to impairing myelination, mitochondrial dysfunction, resulting from an excessive rise in intracellular Ca^2+^ concentrations caused by the over-activation of NMDA receptors, is thought to precipitate white matter damage in hemorrhagic and ischemic stroke (Wang Y. et al., 2016). Indeed, MCU knockdown reduces NMDA-induced excitotoxicity (Qiu et al., 2013).

The astrocyte endfoot is laden with mitochondria that rapidly buffer the large cytosolic Ca^2+^ waves and increase ATP synthesis in support of the massive metabolic requirements for neurovascular coupling (Mulligan and MacVicar, 2004; Grubb et al., 2021; Aten et al., 2022). The importance of mitochondrial function in the astrocyte endfoot is further indicated by a recent study that examined the distribution of glycolytic enzymes and mitochondrial proteins in the cell bodies and endfeet of astrocytes (Stokum et al., 2021). Unlike the cell body that is enriched with glycolytic enzymes, numerous respiratory proteins and at least four subunits of the MCU_*cx*_ (MCUR1, MCU, MICU1, and MICU2) are preferentially located in the astrocyte endfoot (Stokum et al., 2021). The astrocyte endfoot therefore appears to employ the MCU_*cx*_ for neurovascular coupling.

Capillary constriction starts about 1 h after an episode of transient cerebral ischemia (Yemisci et al., 2009; Gursoy-Ozdemir et al., 2012). This increases the risk of brain injury by reducing CBF (Engedal et al., 2018). Ablation of pericytes prevents capillary constriction suggesting that these mural cells mediate reduced CBF after an ischemic stroke (Hartmann et al., 2021). Mitochondrial Ca^2+^ uptake constricts capillaries by increasing ROS production (Hartmann et al., 2021). These findings suggest that the MCU_*cx*_ mediates ischemia-induced capillary constriction by pericytes.

Endothelial cell damage is a prominent feature of ischemic and hemorrhagic brain injury that may also impair CBF by compromising capillary integrity (Zille et al., 2019; Solár et al., 2022). The activation of inducible-nitric oxide synthase in endothelial cells, astrocytes, and microglia by cerebral ischemia produces a massive increase in nitric oxide (Chen Z. Q. et al., 2017). This causes the excessive nitrosylation of mitochondrial proteins and DNA that damages the NVU (Chen Z. Q. et al., 2017). Mitochondrial Ca^2+^ uptake increases the production of nitric oxide by endothelial cells (Dedkova and Blatter, 2005). This finding coupled with evidence of reduced mitochondrial Ca^2+^ uptake and energy production in endothelial cell-specific MCUR1 nulls (Tomar et al., 2016) and vascular dysfunction in mice lacking MICU1 or MICU2 (Hoffman et al., 2013; Bick et al., 2017) suggests that excessive MCU_*cx*_ activity in endothelial cells may contribute to impaired CBF and NVU damage in stroke.

The importance of mitochondria and Ca^2+^ signaling in regulating microglial function is well appreciated (Orihuela et al., 2016; Umpierre and Wu, 2021). The NLRP3 inflammasome is an inflammatory complex that increases production of the injurious pro-inflammatory cytokine IL-1β by microglia and macrophages after an experimental ischemic stroke (Hoffman and Broderick, 2016; Franke et al., 2021). Increased MCU_*cx*_ activity drives activation of the NLRP3 inflammasome (Triantafilou et al., 2013; Dong et al., 2022). Conversely, MCU_*cx*_ inhibition by increased MCUb expression promotes the polarization of macrophages from a pro-inflammatory M1 to an anti-inflammatory M2 phenotype (Feno et al., 2021a). Lastly, MCU ablation restores the phagocytic activity of macrophages with impaired mitochondrial fission (Wang et al., 2017). MCU_*cx*_ inhibition may thus reduce injurious brain inflammation after a stroke by supressing M1 activity and increasing M2 polarization.

### 6.3. MCU_*cx*_ inhibition blocks multiple cell death pathways implicated in ischemic and hemorrhagic damage of the NVU

**FIGURE 5:**
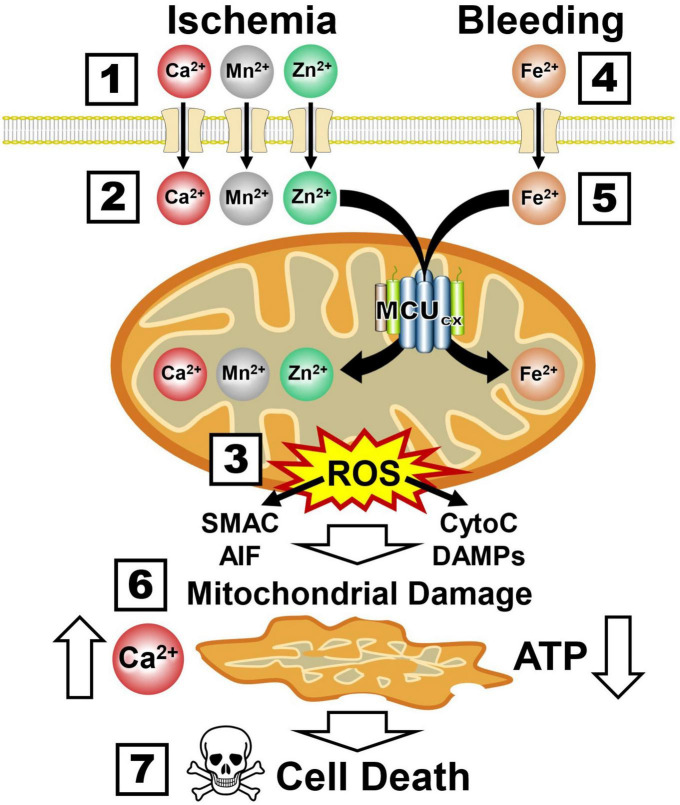
Ca^2+^, Mn^2+^, Zn^2+^, and Fe^2+^ uptake by the MCU_*cx*_ triggers mitochondrial collapse and multiple cell death pathways. Boxes 1–3, Ischemia causes excessive mitochondrial Ca^2+^, Mn^2+^, and Zn^2+^ uptake by the MCU_*cx*_ resulting in injurious ROS overproduction and the release of multiple pro-death factors (SMAC, AIF, CytoC, and DAMPs). Boxes 4–6, Bleeding triggers excessive mitochondrial Fe^2+^ uptake by the MCU_*cx*_ that results in ROS overproduction and the release of pro-death factors (SMAC, AIF, CytoC, and DAMPs). Boxes 7, 8, Mitochondrial collapse impairs Ca^2+^ buffering and ATP synthesis that exacerbates the injurious effects of ROS overproduction and pro-death factor release. (See section “6.3. MCUcx inhibition blocks multiple cell death pathways implicated in ischemic and hemorrhagic damage of the NVU” for abbreviations and mechanistic details).

The ability of mitochondria to rapidly sequester large amounts of Ca^2+^ renders them highly susceptible to injurious mitochondrial Ca^2+^ overloading (Duchen, 2012). In addition to overloading neurons with toxic amounts of Ca^2+^ and Mn^2+^, ischemia also damages neurons by allowing lethal amounts of zinc (Zn^2+^) to enter them by way of Ca^2+^-permeable ionotropic glutamate receptors (Weiss and Sensi, 2000; Figure 5 and Box 1). Like Ca^2+^ and Mn^2+^, the MCU_*cx*_ transports excessive amounts of Zn^2+^ into mitochondria (Giacomello et al., 2007; Medvedeva and Weiss, 2014; Ji et al., 2020; Box 2). This induces excessive ROS production and the release of cytochrome C (CytoC), second mitochondria-derived activator of caspases (SMAC), apoptosis-inducing factor (AIF) and mitochondrial damage-associated molecular patterns (DAMPs) that activate multiple cell death pathways (Green et al., 2014; Feno et al., 2019; Tang and Wu, 2019; Box 3). In hemorrhagic stroke, cerebral blood vessels are ruptured resulting in the release of toxic amounts of ferrous iron (Fe^2+^) from hemoglobin (Xi et al., 2006; Box 4). This triggers excessive Fe^2+^ uptake by the MCU_*cx*_ (Box 5) that induces further ROS over-production and mitochondrial injury (Sripetchwandee et al., 2013, 2014; Box 6). The resultant loss of Ca^2+^ buffering and ATP synthesis (Box 7) exacerbate the death of vascular cells, glia, and neurons that comprise the NVU (Duchen, 2012; Chamorro et al., 2021; Box 8). Since mitochondrial damage is an early pathological event that triggers these diverse cell death pathways (Barsoum et al., 2006; Green et al., 2014), preserving mitochondrial function is an attractive therapeutic approach for stroke.

### 6.4. Protection of the NVU by the inhibition of mitochondrial Ca^2+^ overloading

**FIGURE 6:**
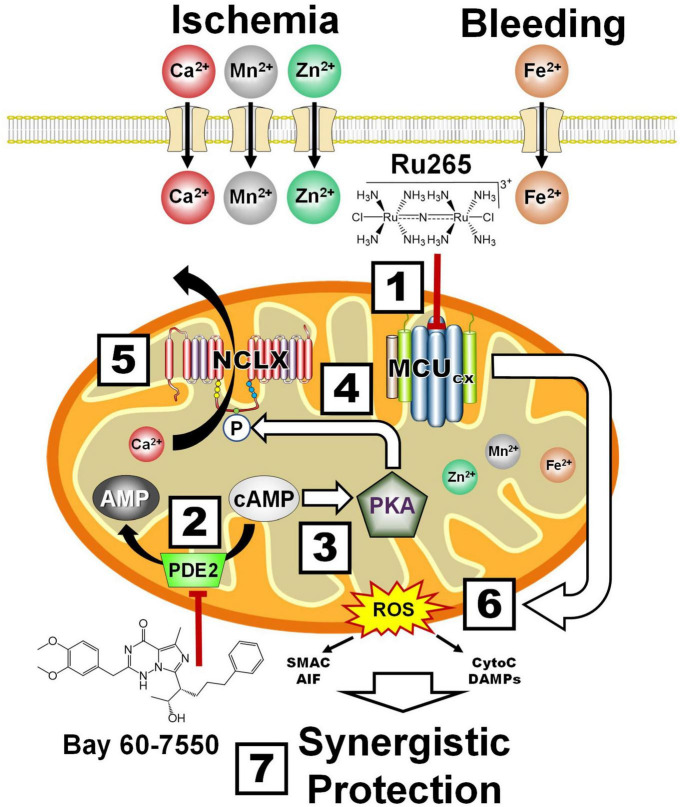
Synergistic protection by combining MCU_*cx*_ inhibition with Ru265 and NCLX activation with Bay 60-7550. Box 1, Ru265 inhibits Ca^2+^, Mn^2+^, Zn^2+^, and Fe^2+^ uptake by the MCU_*cx*_. Box 2, Bay 60-7550 inhibits PDE2. Box 3, The resultant elevation of cAMP levels activates PKA. Box 4, PKA phosphorylates serine residue (S258) of the NCLX. Box 5, This increases NCLX activity resulting in elevated mitochondrial Ca^2+^ efflux. Box 6, MCU_*cx*_ inhibition further reduces mitochondrial Ca^2+^ levels. Box 7, Ru265 and Bay 60-7550 synergistically block ROS overproduction and pro-death factor release that confers profound protection. (See section “6.4. Protection of the NVU by the inhibition of mitochondrial Ca2^+^ overloading” for abbreviations and mechanistic details).

Ru265 is a ruthenium complex (Figure 6 and Box 1) that potently inhibits high-capacity mitochondrial Ca^2+^ uptake by blocking the MCU_*cx*_ (Woods et al., 2019). We have recently shown that Ru265 is highly effective at protecting cortical neuron cultures from damage by a lethal period of OGD (Novorolsky et al., 2020). Although Ru265 reduced sensorimotor deficits and infarct volumes in a mouse model of hypoxic/ischemic brain damage, therapeutic use of Ru265 is limited by poor BBB permeability and seizure induction at high doses (Novorolsky et al., 2020).

Alternatively, mitochondrial Ca^2+^ overloading can be suppressed by increasing the removal of Ca^2+^ from mitochondria. In this regard, we have demonstrated that NCLX mediates mitochondrial Ca^2+^ efflux (Palty et al., 2010). Bay 60-7550 is a neuroprotective compound that potently blocks PDE2 which converts cAMP to AMP (Soares et al., 2017; Figure 6 and Box 2). The resultant elevation of cAMP activates PKA (Box 3). We have demonstrated that PKA activation increases the phosphorylation of serine residue (S258) of the NCLX (Box 4) that markedly enhances mitochondrial Ca^2+^ extrusion (Palty et al., 2010; Kostic et al., 2018; Box 5). By these convergent mechanisms, Ru265 and Bay 60-7550 should synergistically suppress mitochondrial Ca^2+^ overloading, ROS production and the release pro-death factors (Box 6) resulting in profound neural cell protection (Rozenfeld et al., 2022; Box 7). Although this combinatory strategy should allow the use of lower and thus safer doses to protect the NVU, combining Ru265 with Bay 60-7550 may still produce unfavorable drug interactions. Ru265 and PDE2 inhibitors also suffer from adverse side effects cause by unwanted actions on healthy tissues (Baillie et al., 2019). We describe how these limitations can potentially be overcome by encapsulating Ru265 in nanoparticles that readily enter the brain and preferentially target metabolically compromised NVUs.

### 6.5. Activation of nuclear hormone receptors to mobilize diverse cell subtypes necessary for NVU repair

**FIGURE 7:**
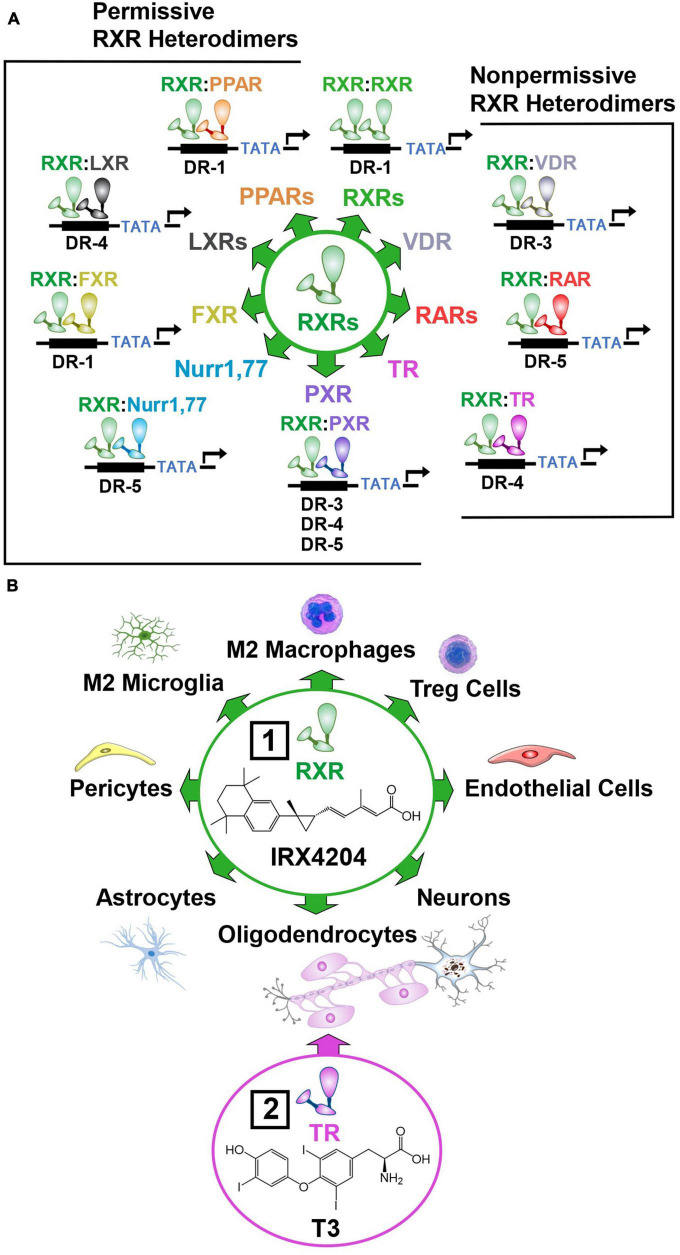
Permissive and non-permissive RXR dimers. In addition to forming homodimers, RXR acts as a universal heterodimerization partner with other nuclear hormone receptors. **(A)** Permissive heterodimers act in a cooperative or synergistic fashion while non-permissive heterodimers generally require ligand binding to the non-RXR monomer to induce transcriptional activity. **(B)** RXRs are ubiquitously expressed and regulate the proliferation, differentiation, function, and survival of numerous cell subtypes in the brain. Box 1, This enables the preferential RXR agonist IRX4204 to reduce inflammation and stimulate the production of new vascular, glial, and neuronal cells necessary for NVU repair. Box 2, The TR agonist T3 enhances the therapeutic benefits of IRX4204 by synergistically inducing the differentiation of oligodendrocyte progenitor cells into myelin producing oligodendrocytes. (See section “6.5. Activation of nuclear hormone receptors to mobilize diverse cell subtypes necessary for NVU repair” for abbreviations and mechanistic details).

To enhance NVU repair, drug targets must be found that mobilize diverse immune, vascular, and neural cell subtypes necessary to restore the intricate architecture and function of this fragile structure (Franklin and Ffrench-Constant, 2017). In this regard, nuclear hormone receptors are particularly promising drug targets (Simandi et al., 2018). Nuclear hormone receptors form dimers that activate a wide array of genes implicated in NVU repair. The RXR plays a central role in these regenerative processes by forming homodimers, and heterodimers with the farnesoid X receptor (FXR), thyroid receptor (TR), vitamin D receptor (VDR), retinoic acid receptor (RAR), pregnane X receptor (PXR), nuclear receptor related 1 and 77 proteins (Nurr1, 77), peroxisome proliferator activated receptor (PPAR), and liver X receptor (LXR) (Simandi et al., 2018; Figure 7A). These RXR dimers bind to the amino acid sequence 5′-RGKTCA-3′ organized as direct repeats with a variable length spacer of 1–5 base pairs (DR1-DR5) that confers DNA binding site specificity (Umesono et al., 1991; Perlmann et al., 1993). RXRs heterodimers have been classified into two categories according to their activation mode. Permissive heterodimers which include RXR/PPAR, RXR/LXR, and RXR/FXR are activated by ligands for either RXR or the binding partner for RXR (Forman et al., 1995). Non-permissive heterodimers such as RXR/RAR, RXR/VDR, and RXR/TR are usually activated only by ligands specific for the RXR partner (Kurokawa et al., 1993). In this case, RXR usually acts as an essential but silent partner (Evans and Mangelsdorf, 2014). By permissive and non-permissive interactions with other nuclear hormone receptors, RXR dimers regulate the complex genomic events that orchestrate NVU repair (Daneman et al., 2010a; Mounier et al., 2015; Simandi et al., 2018; Baldassarro et al., 2019). These events lead to the proliferation of vascular and neural progenitor cells, and the polarization of macrophages, microglia and T cells to pro-repair phenotypes that resolve inflammation, clear cellular debris, and create a fertile environment essential for the differentiation and integration of vascular cells, glia, and neurons into new NVUs that restore brain function (Bi et al., 2010; Chang et al., 2020; Pouso and Cairrao, 2022; Figure 7B).

A key aspect of permissive heterodimers is the cooperative actions of agonists for the RXR and its partner that produce a synergistic response compared to the effects of just a single receptor ligand (Leblanc and Stunnenberg, 1995). Among the so-called non-permissible RXR heterodimers, RXR/TR heterodimers display this cooperative mechanism (Li et al., 2002, 2004; Castillo et al., 2004). This cooperativity allows RXR/TR dimers to increase mitochondrial biogenesis (Weitzel and Iwen, 2011) that enables production of the massive amounts of energy and lipids required for OPC differentiation (Lee and Petratos, 2016; Davies et al., 2021; Figure 7B). In the absence of triiodothyronine (T3), the biologically active form of thyroid hormone, or retinoid acid, mitogen-stimulated rat primary OPC cultures fail to differentiate into oligodendrocytes (Barres et al., 1994). Upon addition of either T3 or retinoic acid to culture medium, OPCs exit the cell cycle and differentiate into oligodendrocytes (Barres et al., 1994). Furthermore, our unpublished findings, described in an expert opinion on therapeutic patents for RXR ligands (Schierle and Merk, 2019), have shown that combining the preferential RXR agonist IRX4204 with T3 synergistically increases the differentiation of OPCs into oligodendrocytes.

### 6.6. RXR agonists: bexarotene and IRX4204

Bexarotene is a clinically approved RXR agonist used to treat cutaneous T cell lymphoma (Duvic et al., 2001). This drug suppresses inflammation, promotes NVU repair, and enhances functional recovery in animal models of ischemic and hemorrhagic stroke (Certo et al., 2015; Xu et al., 2015; Chang et al., 2020; Ting et al., 2020). Several lines of evidence clearly suggest that bexarotene produces these beneficial effects by increasing the production of new mitochondria termed mitochondrial biogenesis (Dickey et al., 2017). The resultant elevation of mitochondrial mass and quality increases maximal respiratory capacity that protects numerous brain cell subtypes (progenitor cells, endothelial cells, pericytes, astrocytes, neurons, and oligodendrocytes) from injury by opposing injurious ROS production (Wu et al., 1999; Scarpulla et al., 2012). A second major benefit of elevated mitochondrial biogenesis is the enhanced production of ATP and lipids essential for neurogenesis, oligodendrogenesis and synaptic plasticity that mediate neurological recovery (Schoenfeld et al., 2010; Schneider et al., 2011; Lees et al., 2019). In support of these restorative actions, bexarotene enhances the dendritic complexity of cultured neurons characterized by increased branching, intersections, and bifurcations (Mounier et al., 2015). The elevation of mitochondrial mass by bexarotene also improves central nervous system (CNS) repair by increasing ATP levels that fuel myelin debris phagocytosis critical for remyelination (Natrajan et al., 2015). Unfortunately, bexarotene may produce adverse cardiovascular side effects that appear to result from the activation of RXR heterodimers with FXR, LXRα, LXRβ, or PPARγ (de Vries-van der Weij et al., 2009; Lalloyer et al., 2009; Paredes et al., 2021) and RARs (Kizaki et al., 1996).

The ability of RXR activation to mobilize diverse brain repair mechanisms resulted in development of the preferential RXR agonist IRX4204 (Figure 7B). IRX4204 is 1000 times more potent at RXRs than RARs and does not activate nuclear hormone receptors implicated in the adverse cardiovascular side effects of bexarotene (Wang J. et al., 2016). IRX4204 reduces paralysis and inflammation in a mouse model of multiple sclerosis called experimental autoimmune encephalomyelitis (Chandraratna et al., 2016). Like bexarotene, IRX4204 also promotes the differentiation of OPCs into myelin-producing oligodendrocytes (Sanders et al., 2014). Based on the efficacy of IRX4204 in several models of Parkinson’s disease (Wang J. et al., 2016), a small clinical trial was conducted to assess the preliminary efficacy and safety of this preferential RXR agonist in individuals afflicted with Parkinson’s disease. This study showed that oral dosing with IRX4204 at 5 mg/day for 14 days was safe and reduced neurological deficits but higher doses (10–20 mg/day) produced hypothyroidism, elevated plasma triglycerides and increased the risk of liver dysfunction (Sanders et al., 2016). These therapeutic limitations can potentially be overcome using nanoparticles that preferentially deliver IRX4204 to damaged NVUs.

### 6.7. TR agonists: triiodothyronine (T3) and thyroxine

In the seminal study by Barres et al. (1994), these investigators showed that injected newborn rats with thyroid hormone increased the number of oligodendrocytes in the optic nerve by over five-fold. Since then, numerous publications have described the ability of T3 or thyroid hormone to promote remyelination after autoimmune-mediated demyelination in the spinal cord (Fernandez et al., 2004; D’Intino et al., 2011), cuprizone-induced demyelination in the corpus collosum (Zhang et al., 2015; Bai et al., 2016; Hartley et al., 2019) and lysolecithin-induced demyelination in the optic chiasm (Payghani et al., 2018). These encouraging findings have led to a Phase I clinical trial that demonstrated liothyronine, a short-acting thyroid hormone, was safe in people with clinically stable MS (Wooliscroft et al., 2020). This encouraging result will hopefully lead to a Phase II clinical trial to further assess the safety and examine the efficacy of liothyronine in MS.

Demyelination of large white matters tracts in the brain is also major pathological feature of ischemic and hemorrhagic stroke (Wang Y. et al., 2016; Tao et al., 2017). Human studies have demonstrated that after cerebral ischemia a reduction of serum T3 is a strong predictor of increased stroke severity and poor clinical outcomes (Alevizaki et al., 2007; Zhang and Meyer, 2010; Jiang et al., 2017). Similarly, low levels of T3 are associated with increased mortality and poor neurological outcomes after a hemorrhagic stroke (Pande et al., 2016). Animal experimentation supports the ability of T3 to promote functional recovery by enhancing remyelination after a hemorrhagic stroke (Vose et al., 2013) as well as neurogenesis, dendritic spine density, and synaptic transmission after an ischemic stroke (Talhada et al., 2019). Only one clinical study to date has examined the effects of boosting T3 levels on neurological outcomes after a stroke (Skvortsova et al., 2006). In this small clinical trial, the administration of thyroid stimulating hormone (thyroliberin), beginning within the first 24 h after an ischemic stroke (*n* = 21), improved functional recovery 21 days later compared to the control group (*n* = 25).

However, T3 has not always been found to promote remyelination. Deletion of myelin regulatory factor results in pervasive CNS demyelination over a 10- to 12-week period followed by gradual but incomplete myelin repair (Hartley et al., 2019). In this genetic model, chronic T3 administration was not tolerated and inhibited OPC proliferation (Hartley et al., 2019). These finding are in keeping with evidence that transient hypothyroidism prior to the differentiation phase enhances remyelination by increasing OPC production (Remaud et al., 2017).

Thyroid hormone produces a variety of adverse side effects such as changes in body weight, sweating, diarrhea, cold intolerance, tachycardia, irregular heart beats, menstrual changes, joint pain, skin rash, and bone loss (He et al., 2020). As a result, individuals taking thyroid medications often experience a reduce quality of life (Peterson et al., 2018). We therefore describe how nanoparticle-based delivery of T3 to the brain can be employed to mitigate the risk of these adverse side effects.

## 7. Nanoparticle-based drug delivery to the brain

Most putative neuroprotective and restorative drugs fail in the clinic because of lack of efficacy (Cheng et al., 2004; Miller, 2010; Choi et al., 2014). Adverse side effects caused by actions on cells outside of the CNS are also a major problem (Cheng et al., 2004; Miller, 2010; Choi et al., 2014). We describe how these problems can potentially be overcome using nanoparticles (NPs) to safely deliver small molecules that protect and repair the NVU. NPs, typically 100 nanometers or less in diameter, loaded with a drug, have been approved as therapeutics by the FDA for over 15 years for various indications (Bobo et al., 2016). NP-based drug delivery for the brain holds tremendous promise as a strategy to improve drug safety by minimizing exposure in healthy parts of the body and maximizing drug concentrations in disease brain tissues (Cheng et al., 2015; Tietjen et al., 2018). However, clinical proof-of-concept for NP-based brain drug delivery remain elusive. Some of the major hurdles include finding the most suitable biocompatible NP building blocks with “intelligent” functionalities such as targeting and ROS sensitive moieties, and tailorable physicochemical properties to optimize NPs for different routes of administration such as intravenous (IV) or intranasal (IN).

### 7.1. Drug delivery to the brain by systemic administration of NPs

Delivery of drugs to the brain after intravascular administration is difficult due to the BBB, which poses both a structural and a metabolic restriction on drug transport and uptake into the brain. In the normal BBB, permeation is highly controlled through transcellular transport mechanisms such as passive diffusion, carrier-mediated transport and various efflux transporters, or transcytosis (receptor-mediated or adsorptive transcytosis (Parrasia et al., 2022). Whereas in diseased states BBB function becomes compromised with temporal as well as regional changes influencing the degree of elevated permeability which, in turn, alter drug delivery.

### 7.2. Implications of compromised BBB integrity for NP-based drug delivery in stroke

Magnetic resonance imaging studies have shown that BBB permeability increases within the first 3 h of onset of an ischemic stroke, continually rises from 6 to 48 h, and remains elevated in the majority of patients 2 months later (Giraud et al., 2015; Merali et al., 2017; Müller et al., 2021). In rodent models of ischemic stroke, BBB permeability increases as early as 25 min after reperfusion and may remain elevated for up to 5 weeks (Strbian et al., 2008; Durukan et al., 2009). In the case of hemorrhagic stroke, computerized tomography imaging studies have shown that BBB permeability is elevated around the hematoma from 24 to 48 h (Lampl et al., 2005; Xu et al., 2017). The time course for BBB disruption in hemorrhagic stroke have been refined by animal studies that indicate the BBB remains largely intact for the first few hours (Yang et al., 1994) but displays integrity loss around the hematoma 6–8 h later (Wagner et al., 1996) that is still evident 5 days later (Jia et al., 2021). For both ischemic and hemorrhagic stroke, increased BBB permeability is associated with unfavorable clinical outcomes (Ivanidze et al., 2018; Nadareishvili et al., 2019). Opening of the BBB likely accounts for the ability of an intracarotid injection of unmodified PLGA-NPs loaded with the antioxidant enzyme superoxide dismutase to reduce brain injury in rats subjected to an ischemic stroke (Reddy and Labhasetwar, 2009). The induction of cell adhesion and signaling molecules on the surface of brain endothelial cells provides a further avenue to enhance the preferential uptake of NPs by compromised NVUs in ischemic and hemorrhagic stroke. In this regard, targeting vascular cell adhesion protein 1 (VCAM-1) and the receptor for advanced glycation end-products (RAGE), overexpressed by endothelial cells subjected to high oxidative stress, has proven to be an effective strategy to increase brain drug delivery in a rat model of ischemic stroke (Liu H. et al., 2016; Kim et al., 2021).

### 7.3. Construction of NPs for systemic drug delivery to the brain: building blocks and functional components

**FIGURE 8:**
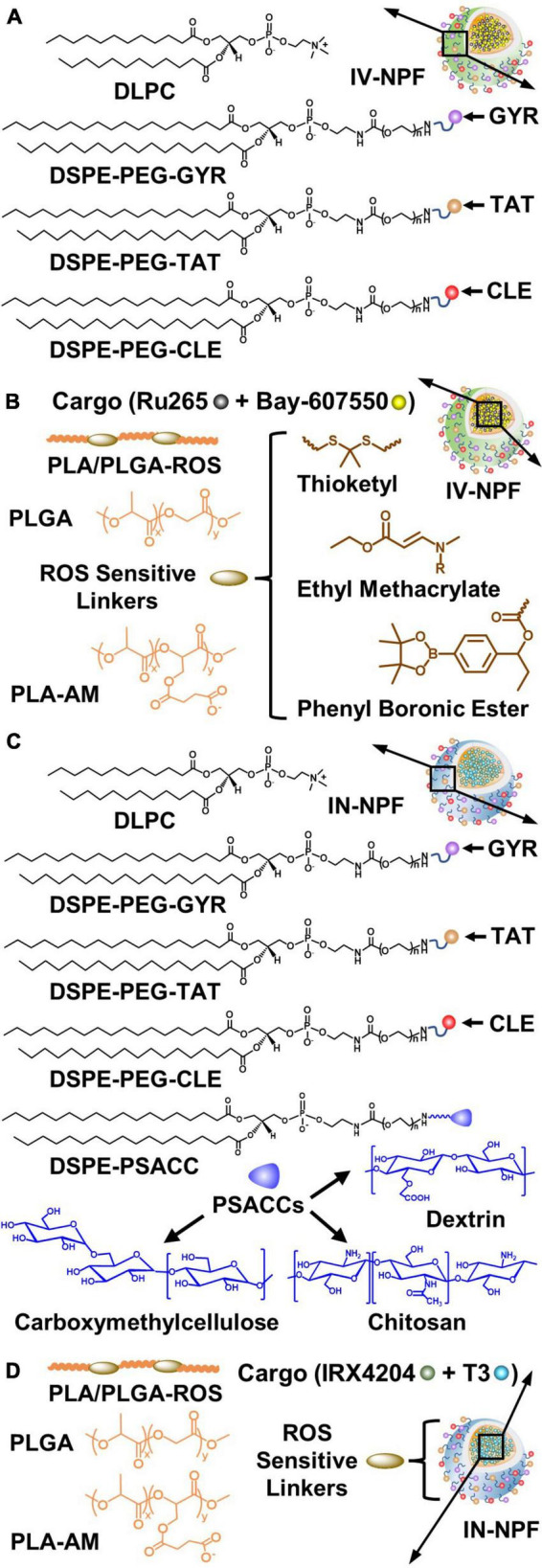
Design strategies for the intravenous (IV) nanoparticle formulation (IV-NPF) and intranasal nanoparticle formulation (IN-NPF) of Ru265 and IRX4204, respectively. **(A)** The IV-NPF shell is comprised of DPLC and DSPE-PEG. The IV-NPF is decorated with GYR, TAT, and CLE by attaching these targeting peptides to DSPE-PEG. **(B)** The IV-NPF core is constructed using polymers of PLA and PLGA. ROS-sensitive linkers such as thioketal, ethyl methacrylate and phenyl boronic ester are incorporated into the PLA/PLGA polymer backbone. **(C)** The IN-NPF shell is also comprised of DPLC and DSPE-PEG-GYR, DSPE-PEG-TAT, and DSPE-PEG-CLE. ROS-sensitive linkers such as thioketal, ethyl methacrylate and phenyl boronic ester are incorporated into the PLA/PLGA polymer backbone. **(D)** The IN-NPF is also coated with low molecular weight PSACC such as dextrin, carboxymethylcellulose, and chitosan to have a long retention time at the nasal mucosal surface and high absorption by nasal epithelia thus minimizing drug exposure in the respiratory system. (See sections “7.3. Construction of NPs for systemic drug delivery to the brain: Building blocks and functional components” and “7.4. Drug delivery to the brain by intranasal administration of NPs” for abbreviations and formulation details).

Nanoparticles with prerequisite physicochemical properties (size <200 nm and positive zeta potential) are typically constructed using polymers of different molecular weights (MW; 0.5–20K) such as poly (lactic acid) (PLA), poly (glycolic acid) (PGA), poly (lactic-co-glycolic acid) (PLGA), and polyethylene glycol (PEG) and lipid-PEGs, or PEG alternatives with reduced immunogenicity (Figure 8A; Pagels and Prud’homme, 2015; Kamaly et al., 2016; Hoang Thi et al., 2020). These polymers have tunable drug release properties, high drug-loading capacity, and excellent safety profiles (Hrkach et al., 2012; Kamaly et al., 2016). Furthermore, conjugated PEG enables the minimal use of formulation stabilizers and decreases the formation of a protein corona on the NP surface that extends systemic circulation of the NP (Gref et al., 2000; Suk et al., 2016). High drug loading can be achieved due to hydrophobic interactions between the neutral drugs (IRX4204 and Bay-607550) and the NP core. By contrast, cationic drugs (Ru265) can be loaded into the NP core by blending with PLA conjugated to anionic moieties (AM; PLA5-20K-AM0.5-5K). To promote preferential and rapid drug release in brain tissues exposed to injurious levels of ROS caused by mitochondrial dysfunction, ROS-sensitive linkers have been incorporated into the PLA and PLGA polymers (PLA/PLGA-ROS) to generate PLA/PLGA5-20K-ROS polymers that selectively degrade when exposed to elevated ROS levels (Figure 8B; Lv et al., 2018; Deng and Liu, 2021).

Despite the strong therapeutic potential of NP drug delivery for stroke, a viable clinical approach has yet to be developed. One strategy that has received considerable attention is the use of cell-penetrating peptides (CPPs) to promote NP uptake and cellular targeting in the brain (Parrasia et al., 2022). Perhaps the best-known member of this family is the TAT protein originally identified in the HIV virus (Frankel and Pabo, 1988; Green and Loewenstein, 1988). A basic region of the TAT protein, termed the protein transduction domain (PTD) that comprises residues 47–57 (YGRKKRRQRRR), is responsible for the ability of TAT to enter cells (Lindsay, 2002; Wadia and Dowdy, 2003). Intraperitoneal injection of mice with TAT-PTD fused to a fluorescence (FITC) or enzyme (β-galactosidase) reporter revealed that all regions of the brain displayed strong labeling at 20 min or 4 h, respectively (Schwarze et al., 1999). NPs coated with the TAT-PTD also show increased brain uptake relative to unmodified NPs (Rao et al., 2008).

Transferrin receptors are attractive targets because these receptors are present on NVU endothelial cells but not endothelial cells in other parts of the body (Jefferies et al., 1984). The GYR peptide (GYRPVHNIRGHWAPG) binds the transferrin receptor (van Rooy et al., 2010). In healthy mice, NPs coated with the GYR peptide are detected in the CNS within 15 min of an IV injection (Wu et al., 2019). Moreover, transferrin receptors on endothelial cells, astrocytes and neurons are upregulated by low oxygen and pro-inflammatory conditions (Dore-Duffy et al., 1993; Du et al., 2012; Wang et al., 2013). In terms of targeting neural cells damaged by an experimental stroke, the CLE peptide (CLEVSRKNC; CLE) preferentially binds to neural cells in damaged brain tissues after an IV injection (Hong et al., 2008; Zhao et al., 2016; Lv et al., 2018).

These CPPs can be attached to the end of 1,2-distearoyl-sn-glycero-3-phosphoethanolamine-N-maleimide PEG (DSPE-PEG2-5K) using maleimide chemistry to produce DSPE-PEG2-5K-TAT, DSPE-PEG2-5K-GYR, and DSPE-PEG2-5K-CLE (Kamaly et al., 2013; Figure 8A). Combining these targeting approaches markedly improves the brain drug delivery and therapeutic efficacy of IV injected NPs in mouse stroke models (Wang et al., 2015; Zhao et al., 2016; Lv et al., 2018). By blending different concentrations of Ru265 and Bay-607550 with varying ratios of PLA/PLGA5-20K-ROS, PLA5-20K-AM0.5-5K, PLGA5-20K, DSPE-PEG2-5K, DSPE-PEG2-5K-TAT, DSPE-PEG2-5K-GYR, and DSPE-PEG2-5K-CLE it is possible to generate NP formulations with different properties such as size, shape, surface charge, kinetics, transport, and toxicity (Hrkach et al., 2012; Mares et al., 2021; Figure 8B).

### 7.4. Drug delivery to the brain by intranasal administration of NPs

Compared to the IV route, the IN route is more convenient and reduces systemic drug exposure (Wang Z. et al., 2019). More importantly, NP formulations customized for nose-to-brain delivery that target the olfactory epithelium (as opposed to the respiratory epithelium) can reach the brain directly, bypassing the BBB, via the olfactory and trigeminal nerves (Gänger and Schindowski, 2018; Wang Z. et al., 2019). Nose-to-brain delivery is a complex process and the exact mechanism is still not well understood (Djupesland et al., 2014). Many different formulation types have been evaluated for IN administration, including liposomes, lipid NPs, hydrogels, dendrimers, and nanoemulsions (Ahmad et al., 2017; Battaglia et al., 2018; Rajput et al., 2022). All these formulations typically contain nano-sized particles made with different biomaterials offering options for the efficient encapsulation of different therapeutic molecules. In addition, unique functionalities such as the use of bioadhesive polysaccharides for the nasal mucosa to increase residence time, permeation enhancers, and thermosensitive gel forming polymeric ingredients have been explored to increase brain delivery.

For example, to increase the retention time at the nasal mucosal surface and enhance absorption by nasal epithelia, NPs have been coated with low MW polysaccharides (PSACC), chitosan, carboxymethylcellulose, and dextrin (Figure 8C). This can be achieved by conjugating the functionalized DSPE head group of DSPE-PSACC2-5K with different MW PSACCs via click chemistry (Figure 8C). Since IN administered NPs also enter the extracellular space in the brain (Gänger and Schindowski, 2018; Wang Z. et al., 2019), NPs designed for IN administration may also be coated with the GYR and TAT peptides to promote endothelial cell uptake and the CLE peptide to target damaged brain tissues (Figure 8C). ROS-sensitive linkers may also be incorporated intro the polymer backbone to promote the selective release of the drug cargo in brain tissues subjected to high oxidative stress. Like the development of NPs for IV administration, blending different concentrations of IRX4204 with varying ratios of PLGA5-20K, DSPE-PSACC2-5K, DSPE-PEG2-5K-GYR, DSPE-PEG2-5K-TAT, and DSPE-PEG2-5K-CLE permits the generation of NPs with distinct properties that can be screened to identify the optimal NP formulation (Figure 8D).

## 8. Overcoming major hurdles in NP-based brain drug delivery

A variety of obstacles for NP-based drug delivery have been elegantly described in several recent reviews (Terstappen et al., 2021; Joseph and Nance, 2022; Nance et al., 2022). Among the most problematic of these include immunogenicity, formation of a protein corona on the NP surface, engulfment of NPs by macrophages in the periphery and microglia in the brain, limited endosomal escape, and toxicity (Saraiva et al., 2016). We describe each of these limitations below and how they may be overcome by creating NP formulations with the optimal size, shape, charge, and surface properties for safely and effectively delivering drugs to the brain. These approaches are designed to maximize NP bioavailability, brain uptake, and safety by producing immune stealth, enhancing cellular transcytosis across the BBB, and maximizing biocompatibility.

### 8.1. PEG alternatives with reduced immunogenic properties

Systemic NPs are often designed with a stealth component using PEG or PEG-conjugated lipids to enhance the circulation time and access to the target site. However, the use of PEG is limited by the immunogenic potential of this polymer resulting in adverse allergic reactions (Kozma et al., 2020). Antibodies (IgG and IgM) against PEG have been detected in about 40% of healthy individuals with no history of treatment with PEGylated therapeutics (Yang and Lai, 2015). This has been attributed to the frequent use of PEG-coupled products in personal care, beauty, and household cleaning products such as soap, sunblock, cosmetics, as well as processed foods (Mohamed et al., 2019). This problem can be overcome by modifying the NP surface to promote immune stealth. Another approach is the use of PEG alternatives with lower immunogenic potential. One such example is poly(β-l-malic acid) (PMLA), a naturally occurring biopolymer with high biocompatibility, biodegradability, and water solubility, as well as low non-immunogenicity (Chi et al., 2016). A further advantage of PMLA is that this polymer has abundant carboxyl groups that can be conjugated with multiple targeting and therapeutic moieties (Chi et al., 2016). This has enabled the creation of PMLA NPs conjugated with peptides that safely and effectively promote transport across the BBB and distribution over multiple brain regions (Israel et al., 2019).

### 8.2. Formation of the protein corona compromises the therapeutic properties of NPs

After exposure to biological fluids, a protein corona rapidly forms on the surface of NPs (Cedervall et al., 2007). Over 300 proteins have been shown to coat NPs (Tenzer et al., 2013; Hacene et al., 2021) that influence their biodistribution, and pharmacokinetic, pharmacodynamic, and toxicological properties (Bertrand et al., 2017; Wang H. et al., 2019). The protein corona also shields targeting peptides on the NP surface and thus prevents them from binding to the intended receptor (Salvati et al., 2013). The protein corona generally can also reduce release of the drug cargo but in some cases may have the opposite effect (Sharifi et al., 2020). The protein corona is also composed of compliment proteins that trigger the removal of NPs by macrophages and may contribute to adverse effects such as allergic responses (Chen F. et al., 2017; Cai et al., 2020). Understanding how the protein corona influences these processes is therefore considered crucial to the development of NPs that safely and effectively enhance drug delivery to the brain.

### 8.3. Modification of the NP protein corona to promote immune stealth and brain uptake

Addition of PEG to the NP surface has been reported to markedly reduce formation of the protein corona by 79% (Schöttler et al., 2016). However, the uptake of PEG-NPs by macrophages was only blocked if PEG-NPs were first incubated with serum proteins (Schöttler et al., 2016). Subsequent analysis revealed that the binding of clusterin to the surface of PEG-NPs was largely responsible for the inhibition of macrophage uptake (Schöttler et al., 2016). PEG therefore appears to confer immune stealth by altering the composition of the protein corona rather than by simply suppressing formation of the protein corona. Moreover, it is possible to direct NPs to the brain by enriching their surface with plasma proteins such as apolipoproteins (ApoA, E, and J). Using liposomes, this was achieved by modifying their surface with a short non-toxic peptide derived from Amyloid β_1–45_ (Amyloid β_25–35_) that specifically interacts with the lipid-binding domain of apolipoproteins (Zhang et al., 2019). This strategy which increases the surface content of apolipoproteins has been shown to enhance the brain delivery and anticancer effectiveness of doxorubicin compared to non-modified liposomes in mice bearing U87 cells (Zhang et al., 2019). Incorporation of Amyloid β_25–35_ has also been reported to have similar benefits for PLGA NPs (Zhang et al., 2019). These finds suggest that modification of the NP surface to encourage the binding of plasma proteins that cross the BBB may be a useful approach to harness the protein corona for targeting drugs to the brain.

## 9. Strategies for overcoming intracellular barriers

### 9.1. Promotion of NP endosomal escape

Depending on the cell type and the composition of the cell surface, NPs can be internalized by clathrin-dependent and clathrin-independent endocytosis (Zhao and Stenzel, 2018). Once inside the cell, NPs either fuse with lysosomes or are recycled back to the cell surface, making endosomal escape a key barrier to delivery of the therapeutic payload (Pichon et al., 2010). One strategy to promote endosomal escape is to incorporate pH-sensitive membrane-disrupting lipids into NPs with amine groups that are ionized in the low pH (5.0–6.5) environment of endosomes. A highly successful example of this approach is the use of ionizable lipids to enhance the endosomal escape of NP-encapsulated mRNA vaccines for COVID-19 (Allen and Cullis, 2013; Cheng et al., 2022). This tactic has also been used to increase the transcytosis of brain-targeting NPs across the BBB. For instance, incorporation of the trileucine endosome escape unit, known to improve the cytoplasmic delivery of NPs (Ding et al., 2011), is an effective method to enhance the brain uptake of CPP-modified NPs (Israel et al., 2019).

### 9.2. Direct translocation of CPPs across the plasma membrane

Recent evidence indicates direct translocation rather than endocytosis mediates the transport of CPPs into the cytosol (Trofimenko et al., 2021; Serulla et al., 2023). The membrane potential provides the driving force for the direct translocation of CPPs (Trofimenko et al., 2021; Serulla et al., 2023). This has been demonstrated by showing that lowering the transmembrane potential boosted the cellular internalization of CPPs (Trofimenko et al., 2021). By contrast, depolarization of the plasma membrane reduces the direct translocation of several CPPs but does not alter their uptake into endosomes (Serulla et al., 2023). Interaction between positively charged CPPs and the negatively charged plasma membrane is thought to lower the membrane potential resulting in a locally megapolarized membrane. This increases the likelihood of water pore formation enabling CPPs to enter cells. The movement of the positive charges carried by the CPPs into the cell, as well as the transport of extracellular cations, dissipates the membrane potential, resulting in the collapse of the water pores and sealing of the plasma membrane. CPP-mediated formation of water pores is thus transient and does not reduce cell viability. This model provides an explanation for how TAT-modified NPs are able to gain direct access to the cytosol (Lin and Alexander-Katz, 2013). Water pores are estimated to be approximately 2 nm in diameter and thus unable to mediate the direct translocation of CPP conjugated proteins or NPs larger than 5 nm (Trofimenko et al., 2021). Nevertheless, harnessing direct translocation offers an approach to avoid the endosomal entrapment of NPs.

## 10. Future perspectives

As described in several recent and comprehensive reviews (Charabati et al., 2020; Joseph and Nance, 2022; Parrasia et al., 2022), many different types of NP formulations have been reported to increase brain drug delivery. Despite these claims, a viable clinical approach has yet to emerge. Several obstacles may account for this failure.

First, there is a demand for therapeutic agents with novel mechanisms of action. More specifically, there is an urgent need for promising drug candidates to treat ischemic and hemorrhagic stroke. To tackle this problem, we have outlined the various neural protective benefits of preventing mitochondria Ca^2+^ overloading by combining MCU inhibition with NCLX stimulation using Ru265 and Bay-607550, respectively. We have also discussed how functional outcomes after a stroke should be further increased by stimulating brain repair through RXR activation with drugs such as bexarotene or IRX4204 and TR agonism with T3. To improve the safety and efficacy of these approved (bexarotene and T3) and investigational (IRX4204) drugs for the treatment of ischemic and hemorrhagic stroke, we have described the use of NP-based drug delivery strategies. For example, NPs can be designed to preferentially deliver their payload to metabolically compromised (vulnerable) or damaged NVUs. Furthermore, we have discussed how combining the rapid IV administration of NPs containing Ru265 and Bay-607550 with the repeated IN delivery of NPs loaded with IRX4204 and T3, should achieve positive clinical outcomes in the treatment of ischemic and hemorrhagic stroke by protecting vulnerable and repairing damaged NVUs.

Second, the development of NP-based drug products has its own challenges and complexity. However, the remarkable success of NP-based vaccines in combating the recent COVID-19 pandemic has demonstrated that the large-scale production of NPs is an achievable goal. During the development of neuroprotective and restorative treatments for stroke, it will be crucial to employ rigorous preclinical testing guidelines for effective translational research in stroke (Lapchak et al., 2013). Furthermore, systematic studies comparing and correlating the physiochemical properties, safety, and efficacy of the various NP-based approaches are necessary to understand the mechanism of enhancement of brain drug delivery and select NP compositions with the best performance. Such benchmarking studies are essential for identification of the most promising candidates for clinical testing but are unlikely to be performed by academic laboratories that have limited resources. Resolution of these problems will therefore require rigorous standardization procedures, diverse technical skills, extensive manufacturing capabilities, and large testing facilities.

Third, drug development for neurodegenerative disorders still suffers from an exceptional high failure rate. Hence, major biotechnology companies and pharmaceutical firms are reluctant to commit the considerable resources required for such risky drug discovery efforts. Nevertheless, we have described how recent innovations in targeting mitochondrial Ca^2+^ handling and nuclear hormone receptors with small molecules and preferentially delivering them to where they are required in the brain may very well tip the risk-benefit balance toward renewed investment in therapeutic development for ischemic and hemorrhagic stroke.

## Author contributions

All authors contributed to the article, agreed to be accountable for the content of this work, and approved the submitted version.
